# Mechanistic insights into the activation of the IKK kinase complex by the Kaposi’s sarcoma herpes virus oncoprotein vFLIP

**DOI:** 10.1016/j.jbc.2022.102012

**Published:** 2022-05-05

**Authors:** Claire Bagnéris, Swathi L. Senthil Kumar, Mehdi Baratchian, Hannah M. Britt, Tufa E. Assafa, Konstantinos Thalassinos, Mary K. Collins, Tracey E. Barrett

**Affiliations:** 1 Department of Biological Sciences, Institute of Structural Molecular Biology, Birkbeck College, London, UK; 2 Genitourinary Malignancies Research Center, Lerner Research Institute, Cleveland Clinic, Cleveland, Ohio, USA; 3 Division of Biosciences, Institute of Structural and Molecular Biology, University College London, London, UK; 4 Chemistry and Biochemistry Department, University of California Santa Cruz, Santa Cruz, California, USA; 5 Okinawa Institute of Science and Technology, Graduate University, Onna-son, Okinawa, Japan

**Keywords:** KSHV, canonical NF-kappaB pathway, vFLIP, IKK kinase, IKKgamma, IKKbeta, constitutive activation, EPR, electron paramagnetic resonance, IKK, inhibitor of κB kinase, KSHV, Kaposi’s sarcoma-associated herpes virus, LPP, lambda phosphatase, MALS, multi angle light scattering, SEC, size-exclusion chromatography, TNFα, tumor necrosis factor alpha

## Abstract

Constitutive activation of the canonical NF-κB signaling pathway is a major factor in Kaposi’s sarcoma-associated herpes virus pathogenesis where it is essential for the survival of primary effusion lymphoma. Central to this process is persistent upregulation of the inhibitor of κB kinase (IKK) complex by the virally encoded oncoprotein vFLIP. Although the physical interaction between vFLIP and the IKK kinase regulatory component essential for persistent activation, IKKγ, has been well characterized, it remains unclear how the kinase subunits are rendered active mechanistically. Using a combination of cell-based assays, biophysical techniques, and structural biology, we demonstrate here that vFLIP alone is sufficient to activate the IKK kinase complex. Furthermore, we identify weakly stabilized, high molecular weight vFLIP–IKKγ assemblies that are key to the activation process. Taken together, our results are the first to reveal that vFLIP-induced NF-κB activation pivots on the formation of structurally specific vFLIP–IKKγ multimers which have an important role in rendering the kinase subunits active through a process of autophosphorylation. This mechanism of NF-κB activation is in contrast to those utilized by endogenous cytokines and cellular FLIP homologues.

The Kaposi’s sarcoma-associated herpes virus (KSHV) is the main etiological agent of Kaposi’s sarcoma and the lymphoproliferative disorder primary effusion lymphoma (1, 2, 3, 4). Pivotal to the survival of both tumors is the canonical NF-κB pathway that becomes constitutively activated by the virally encoded protein vFLIP (5, 6, 7). It has been shown that vFLIP subverts the normally tightly regulated IKK kinase complex that is central to upregulation of the pathway.

The canonical NF-κB pathway is dependent on the transcription factors RelA, p65, Rel-c, and p50 that operate as heterodimers or homodimers (8, 9) and are normally sequestered in the cytoplasm through associations with members of the IκB family of inhibitory proteins. These interactions result in the occlusion of their nuclear localization sequences and κB binding motifs, thus preventing both translocation to the nucleus and gene regulation. Activation of the pathway therefore requires disruption of the inhibitory complexes in a process initiated by the IKK kinase complex, a multisubunit ∼700 kDa assembly that transiently includes the chaperone and co-chaperone Hsp90 and Cdc37, respectively (9, 10). The core enzyme, however, comprises the kinase subunits IKKβ and/or IKKα and the essential nonenzymatic regulatory subunit IKKγ or Nemo. Although IKKα has been shown to be a participant, the main kinase subunit is IKKβ whose phosphorylation is crucial for activation of the canonical pathway (11). Following formation of an activated kinase, the IκB inhibitory proteins are targeted for phosphorylation as a precursor to K48-linked polyubiquitination and degradation by the 26S proteosome enabling the transcription factors to transition to the nucleus and associate with their cognate κB motifs.

Activation of the IKK kinase complex in response to signals transduced by cytokines such as tumor necrosis factor alpha (TNFα) *via* upstream receptors requires the association of IKKγ with K63-linked or linear ubiquitin chains, followed by phosphorylation of the IKKβ subunits on their activation or T-loops by the kinase TAK1 (12). Mutation of serines S177 and S181 within this region to alanine has been shown to abolish activation of the kinase complex in response to TNFα, whilst replacement with phosphomimetic glutamates renders it constitutively active (7).

In contrast to the ubiquitin-dependent mechanisms, persistent IKK kinase activation by vFLIP proceeds *via* a direct association between vFLIP and IKKγ (9, 13, 14). Structural and mechanistic studies have enabled detailed characterization of the vFLIP–IKKγ complex (15, 16, 17); however, the way in which vFLIP activates the kinase subunits has remained elusive. This is owing to the fact that the vFLIP and kinase binding sites within IKKγ are separated by approximately 160 amino acid residues, and so far, it has only been established that there appears to be no involvement of IKKγ residues C terminal to 254 (13, 14). Although conformational changes within IKKγ induced by vFLIP could be envisaged to bring both sites into proximity as part of an activation mechanism, none have been observed to date (17, 18). Similarly, the participation of upstream activators such as the kinase TAK1 and the linear ubiquitin conjugating complex LUBAC are not required for vFLIP-induced IKK kinase activation (14, 19).

Based on observations that at high concentrations, IKKα and IKKβ can initiate a process of autophosphorylation through the generation of higher order assemblies (20, 21), we investigated whether activation of the kinase complex could result solely from the association of vFLIP and IKKγ. We also explored the potential involvement of higher order vFLIP–IKKγ complexes given that lattice structures consistent with oligomeric IKK kinase assemblies have been observed by super-resolution microscopy in unstimulated U2OS cells (22). Using a combination of biochemical/biophysical, cell-based, and structural approaches, we have established that vFLIP directly induces autophosphorylation of IKKβ in a mechanism dependent on higher order vFLIP–IKKγ assemblies.

## Results

### Activation of the IKK kinase complex *in vitro* can be achieved with vFLIP alone and derives from autophosphorylation of IKKβ

**Figure 1:**
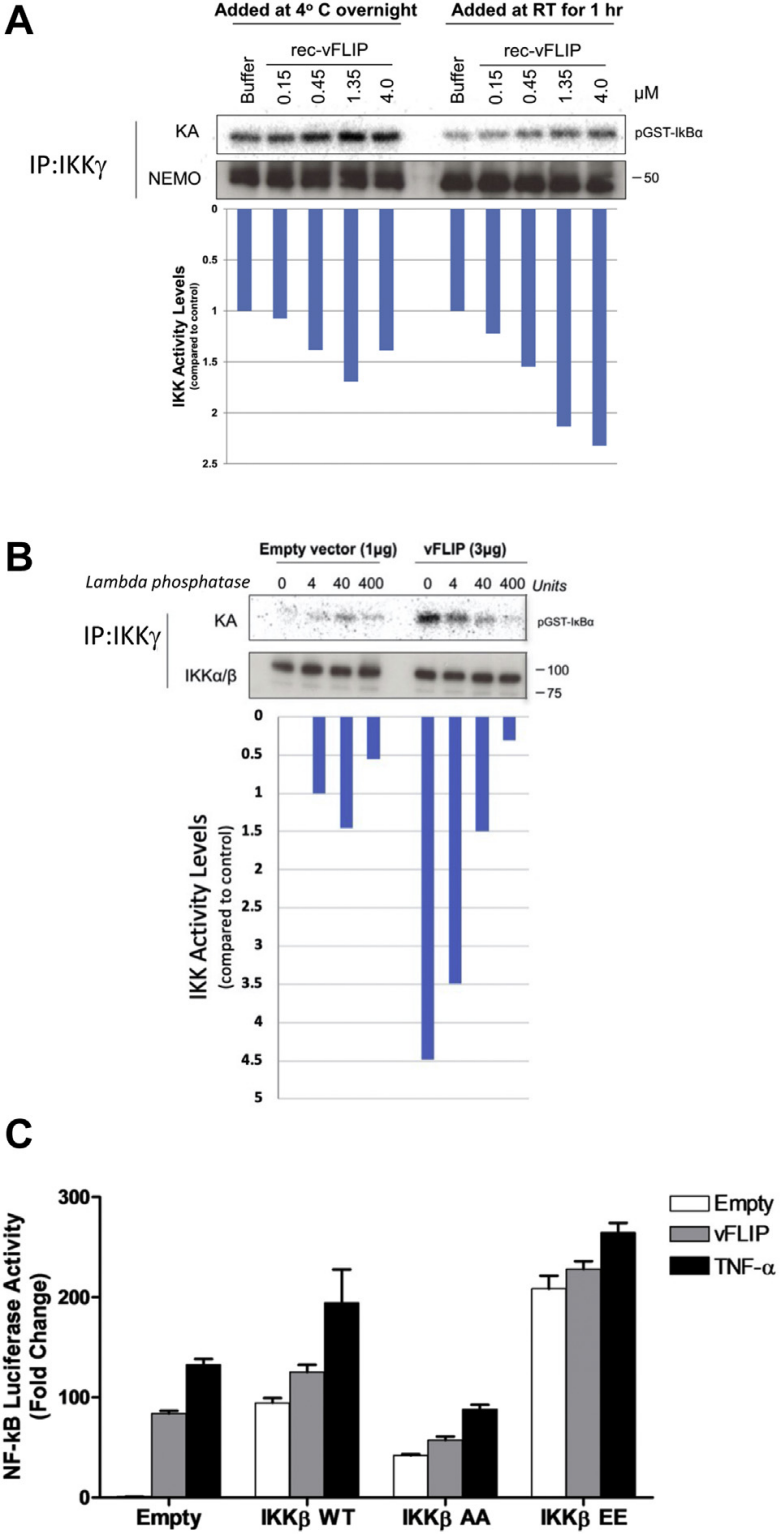
**Cell-based assays to establish whether recombinant vFLIP can activate the IKK kinase complex and phosphorylate recombinant GST-IκBα (1–54).***A*, immunoprecipitates of the IKK kinase complex from unstimulated HEK293T cell lysates (obtained using an anti-IKKγ antibody) were incubated with increasing concentrations of recombinant vFLIP (0.15–4.0 μM) for 1 h at room temperature or at 4 °C overnight, followed by a kinase assay (KA) to assess their capacity to phosphorylate recombinant GST-IκBα (1–54) (pGST-IκBα) in the presence of γ^32^p-ATP. *B*, kinase activity assay of IKK kinase complex immunoprecipitates from lysates of HEK293T cells transduced with vFLIP that had been treated with lambda phosphatase prior to analysis. *C*, HEK293T cells that had been transiently transfected with empty vector or pCDNA3-vFLIP in combination with WT IKKβ or the IKKβ mutants IKKβ AA (S177/S181AA) and IKKβ EE (S177/S181EE) were analyzed for NF-κB activity using a luciferase NF-κB reporter system and compared to those lacking vFLIP, but alternatively stimulated with TNFα. These are technical replicates where the mean ± standard deviation is shown. The experiment shown is one of four biological replicates, all giving similar results. IKK, inhibitor of κB kinase.

Using knockdown cell lines, we and others have previously demonstrated that vFLIP does not require the upstream kinases TAK1 or MEKK3 to activate the IKK kinase complex (14, 19). We have further demonstrated that recombinant vFLIP added to cell lysates can activate the IKK kinase resulting in phosphorylation of IκBα (14) and that a direct association with IKKγ is required based on our studies involving an IKKγ mimetic, stapled peptide competitive inhibitor (16). This is in contrast to recombinant p22-cFLIP that fails to activate the IKK kinase consistent with a dependency on TAK1 (14). Based on this inability to identify coactivators for vFLIP-mediated NF-κB activation coupled with the fact that only Hsp90 and Cdc37 were observed to copurify with the vFLIP–IKK kinase complex (9, 14, 19), we sought to determine whether activation of the kinase could be specifically linked to vFLIP binding. To investigate this, we purified the IKK kinase complex from unstimulated HEK293T cell lysates using an anti-IKKγ antibody followed by incubation with increasing concentrations of recombinant vFLIP. These studies were initially conducted at room temperature for 1 h and were followed by a kinase assay to determine the capacity of the resulting vFLIP–IKKγ assemblies to phosphorylate recombinant GST-IκBα (1–54). Since rapid dose-dependent activation was observed, the same assays were conducted at 4 °C for comparison where a similar trend was evident but with lower levels of activation consistent with reduced temperature (Fig. 1*A*).

To verify the link between vFLIP and the phosphorylation of GST-IκBα in our assays, experiments were also conducted using lambda phosphatase (LPP). This was because attempts to detect phosphorylation of GST-IκBα(1–54) using immune-precipitated IKK kinase failed to produce a detectable signal, most likely due to limited protein concentration. Samples of immunoprecipitated IKK kinase complexes from HEK293T cells transduced with vFLIP were therefore incubated with LPP prior to analysis in order to establish whether dephosphorylation attenuated kinase activation (Fig. 1*B*). Treatment of the immunoprecipitates with LPP lead to a substantial decrease in activity that was inversely correlated to LPP concentration. However, LPP is a nonspecific phosphatase and thus has the potential to dephosphorylate as yet uncharacterized phosphoacceptors in IKKβ or IKKγ that could also have important roles in vFLIP-induced activation. We therefore examined whether phosphorylation of the IKKβ T-loop was specifically required for upregulation. Although this is an essential prerequisite for NF-κB activation by proinflammatory stimuli, other mechanisms leading to constitutive kinase activation have also been reported that involve residues outside of the T-loop region (23). The capacity of vFLIP to specifically induce phosphorylation of IKKβ T-loop serine mutants, previously shown to either reduce kinase activity (IKKβ S177/S181AA, IKKβ AA) or promote persistent activation (IKKβ S177/S181EE, IKKβ EE (7)) as a control, was therefore determined (Fig. 1*C*). Similar to stimulation with TNFα, vFLIP-mediated NF-κB activation was reduced, when vFLIP was co-expressed with the IKKβ AA mutant. In contrast, high levels of constitutive activation independent of vFLIP were observed for the IKKβ EE phosphomimetic. Taken together, our results confirm that vFLIP-induced activation of the IKK kinase complex is not only direct but also requires the specific phosphorylation of T-loop serines within IKKβ *via* a process of autophosphorylation.

### The identification of higher order vFLIP–IKKγ assemblies

**Figure 2:**
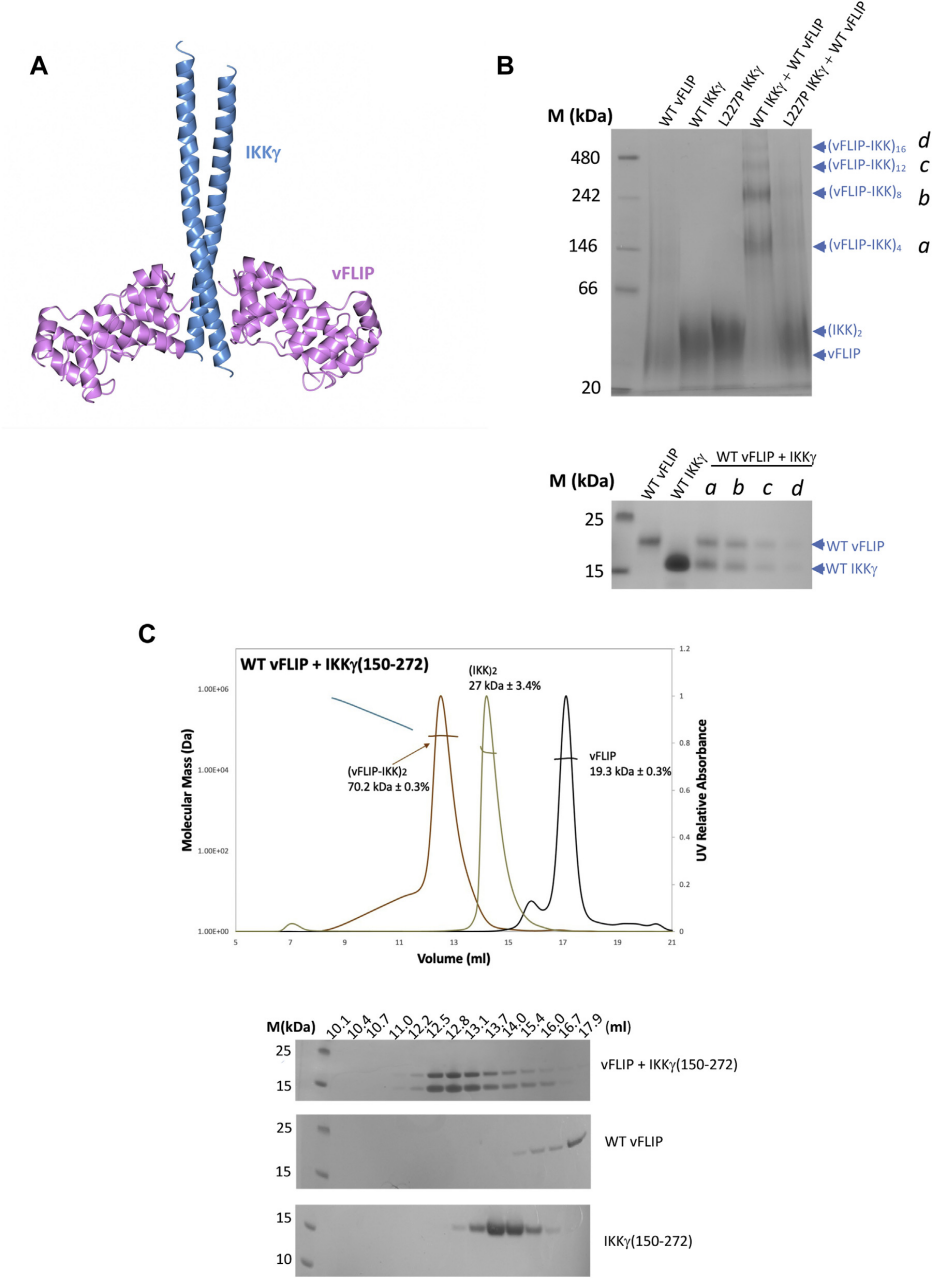
**The identification of higher order vFLIP–IKKγ species.***A*, *cartoon* depiction of the published vFLIP–IKKγ heterotetramer complex (PDB accession code 3CL3). IKKγ is shown in *blue* and vFLIP in *pink*. *B*, *top*, blue native gel analysis of vFLIP, WT/L227P IKKγ, and associated vFLIP–IKKγ complexes. *Bottom*, SDS PAGE analysis of proteins extracted from bands taken from the WT vFLIP and IKKγ lanes of the blue native gel shown in the *top panel*, together with those corresponding to the shifted species (labeled a–d) from the WT vFLIP–IKKγ(150–272) lane. *C*, *top*, SEC MALS analysis of the WT vFLIP–IKKγ(150–272) complex (*brown*) together with those for IKKγ (*khaki*) and vFLIP (*black*) for comparison. For the WT vFLIP–IKKγ(150–272) complex, the predominant peak corresponds to a heterotetramer (2:2 vFLIP:IKKγ) of approximate molecular weight ∼70 kDa (*red line*), whilst the broader, upstream peak or “shoulder” corresponds to a polydispersed mixture of higher molecular weight species (*cyan line*). *Bottom*, SDS PAGE analysis of the elution peaks for vFLIP, IKKγ, and the vFLIP–IKKγ complex. All lanes marked M in the gels shown in (*B*) and (*C*) correspond to those containing molecular weight markers. All experiments were performed in triplicate. IKK, inhibitor of κB kinase.

**Figure 3:**
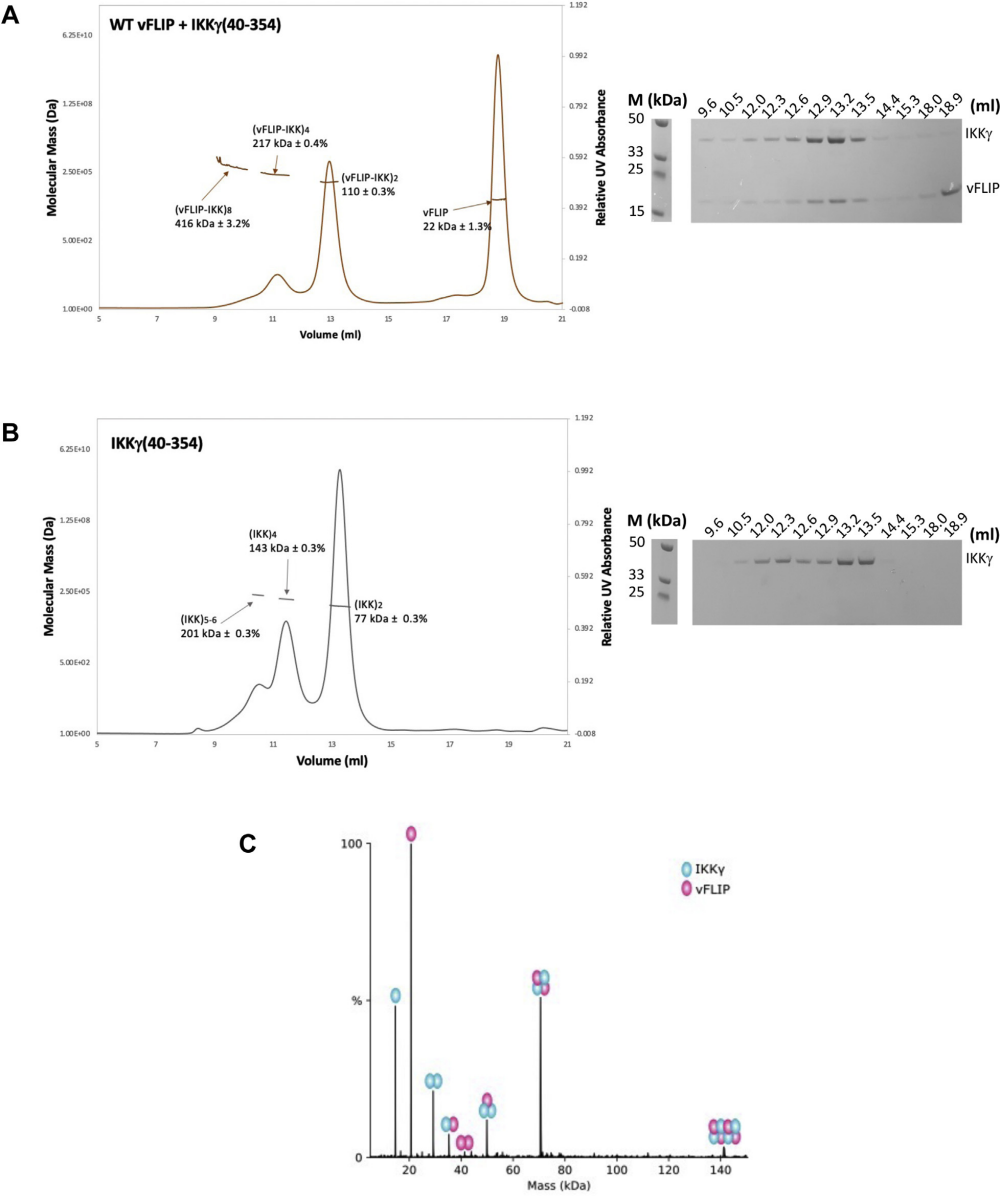
**Quantification of the vFLIP–IKKγ higher order species.***A*, *top left*, SEC MALS trace obtained for the WT vFLIP–IKKγ(40–354) complex revealing the presence of tetrameric and octameric species. *Top right*, SDS PAGE analysis of the eluted fractions. *B*, the equivalent trace and SDS PAGE analysis for IKKγ(40–354) alone. Molecular weight marker lanes are denoted by M in (*A*) and (*B*). *C*, deconvoluted native mass spectrum obtained for the WT vFLIP–IKKγ(150–272) complex. All experiments were performed in triplicate. IKK, inhibitor of κB kinase; SEC, size-exclusion chromatography.

Having established that vFLIP alone is sufficient for activation of the IKK kinase by means of a mechanism involving autophosphorylation of essential T-loop serines in IKKβ, we next focused on how this might be achieved. Since oligomerization of IKKα/β had been previously implicated in autophosphorylation, we explored the possibility that multimeric vFLIP–IKKγ assemblies might be generated leading to autophosphorylation of the kinase subunits. Given that the capacity of size-exclusion chromatography (SEC) to disrupt weak complexes (affinities in the micromolar range or below) has been well documented, we initially sought to ascertain whether discrete, higher order vFLIP–IKKγ complexes could be identified using blue native gel electrophoresis and the IKKγ(150–272) construct used in the original structural studies ((15), Fig. 2, *A* and *B*). The combination of vFLIP and IKKγ(150–272) resulted in several shifted species indicating the existence of oligomeric assemblies. This is in contrast to vFLIP combined with the L227PIKKγ(150–272) mutant which has been linked to the genetic disorder ectodermal dysplasia with immunodeficiency (24, 25, 26), where oligomerization is significantly impaired. The L227P mutant has been shown to attenuate cytokine-mediated NF-κB activation through destabilizing the structure of IKKγ in the vicinity of the vFLIP binding site (15, 27), and our results indicated that the observed oligomerization was unlikely to be an artefact owing to its dependence on correctly folded IKKγ. This was also verified by SDS-PAGE analysis of the shifted bands which confirmed the presence of equivalent quantities of vFLIP and IKKγ in each (Fig. 2*B*, bottom). Analysis of the sizes of these shifted species in the WT vFLIP–IKKγ(150–272) complex revealed that the most intense band had an approximate molecular weight just below 146 kDa, consistent with a hetero-octameric arrangement (4:4 ratio of vFLIP to IKKγ), accompanied by weaker bands representative of 16mer and higher order complexes. Interestingly, there appeared to be little evidence of the heterotetramer that would have a molecular weight of ∼70 kDa. We also analyzed the vFLIP–IKKγ(150–272) complex using SEC MALS (multi angle light scattering). Elution profiles revealed a heterotetramer complex (2:2 ratio of vFLIP to IKKγ) to be the predominant species (70.2 kDa, Fig. 2*C*), along with a much less abundant, higher molecular weight broad peak or “shoulder” indicative of a heterogeneous mixture of oligomeric states. The presence of these low-level higher order species in SEC MALS experiments prompted us to investigate whether these could be better resolved. We therefore conducted analogous experiments with the longer IKKγ construct (IKKγ(40–354)) used in our reported electron paramagnetic resonance (EPR) studies ((18), Fig. 3, *A* and *B*). In contrast to those performed with IKKγ(150–272), a well-defined peak consistent with a hetero-octameric vFLIP-IKKγ assembly (217 kDa), along with a “shoulder” indicative of a mixture of interchanging oligomerization states with an average molecular weight of 416 kDa and therefore a 16mer assembly, were observed together with the heterotetramer (Fig. 3*A*).

To further investigate the existence of vFLIP–IKKγ oligomers, we also analyzed vFLIP–IKKγ complexes using native mass spectrometry involving the shorter IKKγ(150–272) construct (Fig. 3*C*) since attempts with those assembled using IKKγ(40–354) were unsuccessful. These results revealed that whilst the heterotetrameric 2:2 vFLIP:IKKγ complex was again the most predominant species, hetero-octameric and higher order complexes (8:8, Fig. S1*A*) could also be identified in agreement with those observed in blue native gels and SEC MALS experiments involving the longer construct. Further analysis revealed that vFLIP is more loosely associated than IKKγ. Dissociation of the octameric species using collision-induced dissociation (CID, Fig. S1*B*) generated two distinct heptamers, the more predominant resulting from loss of a single vFLIP subunit and the less abundant, loss of an IKKγ monomer. This is consistent with the presence of a 1:2 vFLIP:IKKγ complex both in the original spectrum containing all species (Fig. S1*A*) and in the CID spectrum obtained for the heterotetramer (Fig. S1*C*). These results are in agreement with the crystal structure of the heterotetramer complex (PDB accession code 3CL3) where IKKγ adopts a dimeric coiled-coil configuration in contrast to vFLIP whose only interactions are with IKKγ in the assembly (Fig. 2*A*). The 1:2 vFLIP–IKKγ trimer most likely originates from dissociation of the higher order assemblies under the conditions used. SEC experiments conducted in the buffers used for mass spectrometry (data not shown) revealed that vFLIP–IKKγ complexes are considerably more prone to precipitation under these conditions and are thus less stable. This reduction in stability, due to the buffers used, would explain the greater abundance of higher order complexes observed in the blue native gels and SEC MALS studies relative to the native mass spectra. There is also evidence that the higher order assemblies are intrinsically more susceptible to dissociation given the higher proportions of monomeric vFLIP and IKKγ observed in the CID spectrum of the hetero-octamer compared to the heterotetramer (Fig. S1, *B* and *C*), both spectra having been generated under identical conditions. Interestingly, native mass spectrometry also revealed that IKKγ and vFLIP appear to exist in both folded and partially unfolded states. This is evident in mass spectra of the individual proteins (Fig. S2, *A* and *B*), in which at least two distinct charge state distributions are observed for each monomeric species. Since it is known that the observed charge state in native mass spectrometry is determined by the number of solvent exposed basic residues (28), it is possible to attribute the low charge distributions (m/z > approximately 1700) to folded structures and the more highly charged distributions (m/z < approximately 1700) to partial unfolding. A similar mix of distributions, indicating folded and partially unfolded states, is observed for both monomeric proteins in the vFLIP–IKKγ assembly spectrum (Fig. S1*A*). Conformational plasticity in IKKγ has also been reported by other groups using alternative biophysical and biochemical techniques (27, 29, 30).

**Figure 4:**
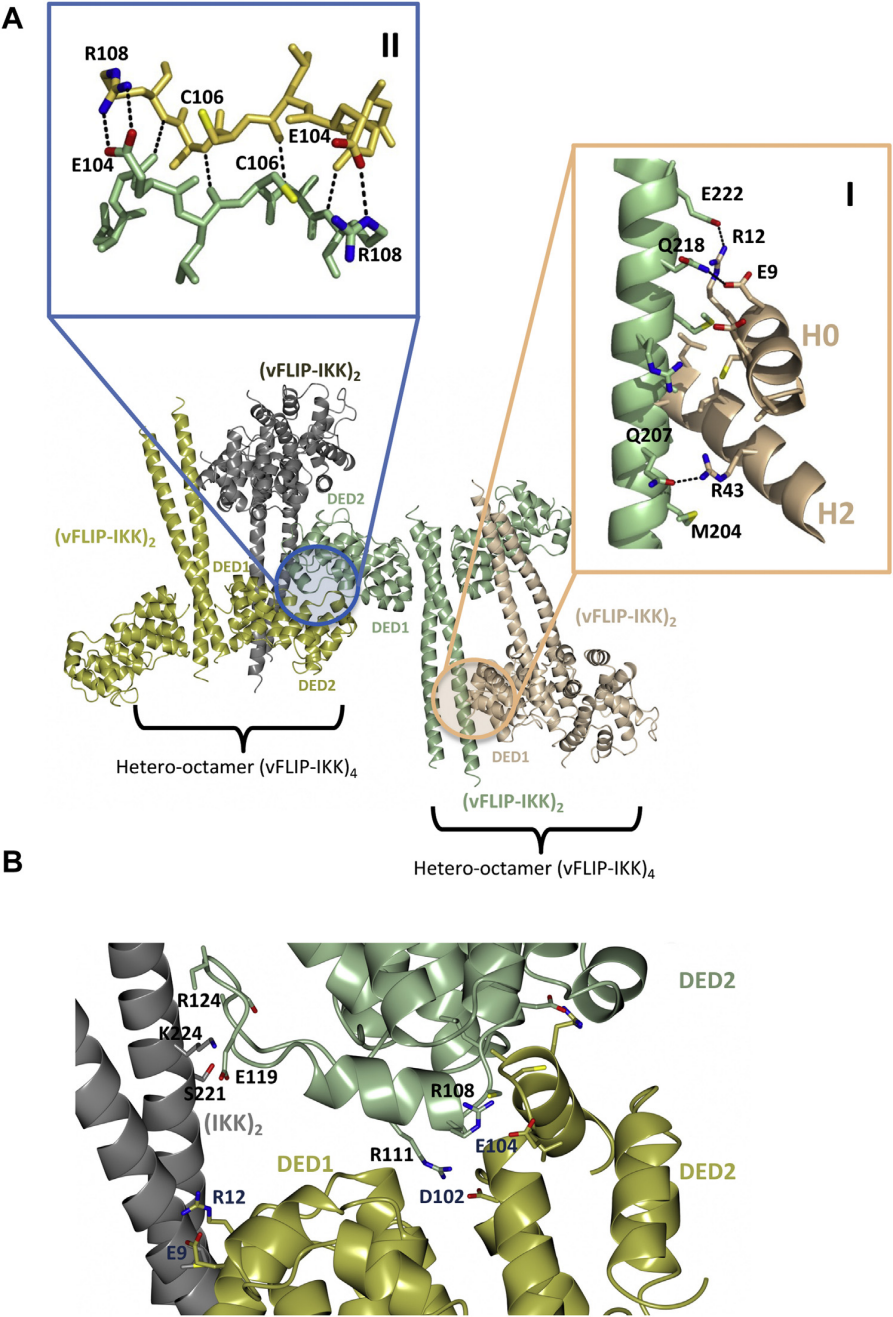
**The identification of oligomeric interfaces in the reported vFLIP–IKKγ structure (PDB accession code****3CL3****) based on an analysis of crystal packing.***A*, *cartoon* depiction of the higher order assemblies arising from vFLIP–IKKγ heterotetramers colored *gold*, *gray*, *light green*, and *wheat*. The vFLIP–IKKγ and vFLIP–vFLIP interfaces have been highlighted in insets I and II, respectively. Inset I, a hetero-octameric vFLIP–IKKγ arrangement is stabilized by residues spanning 204 to 222 of IKKγ (*light green*) and those located within helices H0 and H2 of DED1 in vFLIP (*wheat*). Inset II, vFLIP–vFLIP interactions link the hetero-octamers through the formation of a two-stranded β-sheet that derives from main chain hydrogen bonds involving residues E104 to R108 (*green* and *gold*). This configuration is further stabilized by salt bridges between the guanidinium moieties and carboxylate groups of these residues. *B*, figure illustrating how the vFLIP–IKKγ and vFLIP–vFLIP regions in the hetero-octamer are spatially connected resulting in a continuous interface encompassing DED1 and DED2 interactions in adjacent vFLIP monomers (*gold and green*). DED, death effector domain; IKK, inhibitor of κB kinase.

Following verification of the existence of higher order species, we revisited the original vFLIP–IKKγ(150–272) crystal structure to determine whether stable octameric or higher order species could be identified, based on an analysis of crystal packing, and interfaces that could be investigated using site directed mutagenesis. We were not only able to identify a vFLIP–IKKγ hetero-octamer but also the propensity of this octamer to form higher order assemblies arising from the association of participating heterotetramers (Fig. 4). These putative oligomers appeared to be stabilized through a combination of vFLIP–IKKγ and vFLIP–vFLIP contacts distant from the main vFLIP–IKKγ interface of the heterotetramer. Analysis using the PISA server (https://www.ebi.ac.uk/pdbe/pisa/) revealed that formation of the vFLIP–IKKγ octamer buries approximately 1820 Å^2^ of solvent accessible surface area consistent with a stable, biologically significant assembly, although more weakly stabilized than the heterotetramer (2:2 vFLIP:IKKγ complex) that buries 5420 Å^2^.

Inspection of the octamer interface reveals that key to stabilization are interactions mediated by residues 204 to 222 in IKKγ and helices H0 and H2 in death effector domain 1 of vFLIP (Fig. 4*A*, Inset I). The core of the vFLIP–IKKγ interface involves largely hydrophobic contacts encircled by electrostatic interactions. Key contributors to the electrostatic contacts are a salt bridge involving the carboxylate and guanidinium head groups of R12 in vFLIP and E222 in IKKγ respectively, along with a hydrogen bond donated by the amide nitrogen of Q218 to the carboxylate group of E9. The octamer is further stabilized by a hydrogen bond mediated by the guanidinium head group of R43 and the amide side chain of Q207. By contrast, the vFLIP–vFLIP interface that connects hetero-octamers is largely electrostatic and mediated by residues E104 to R108 located in the region between helices H1 and H2 in death effector domain 2 (Fig. 4*A*, Inset II). Residues in this region form a short two stranded β-sheet that is additionally stabilized by salt bridges involving E104 and R108. Two of the eight vFLIP monomers in the vFLIP–IKKγ 16mer (Fig. 4*A*) contribute both vFLIP–IKKγ and vFLIP–vFLIP contacts thus connecting the two interfacial regions (Fig. 4*B*). In this configuration, the loop spanning residues E119-R124 in one of the vFLIP subunits interacts with an IKKγ monomer through a small number of nonspecific van der Waals contacts and electrostatic interactions involving residues S221 and K224.

### Mutants designed to disrupt vFLIP–IKKγ oligomerization potently downregulate NF-κB activation

**Figure 5:**
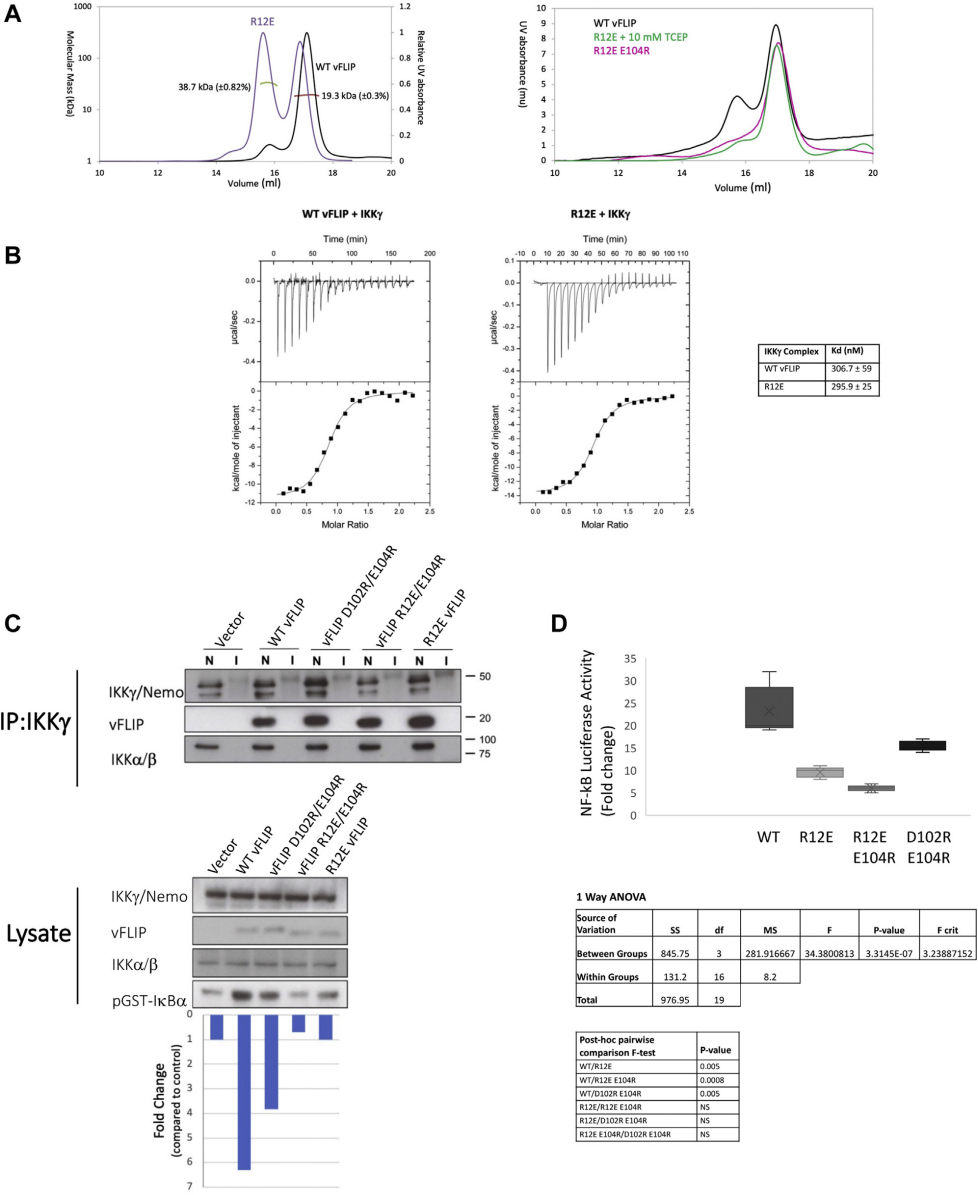
**Biophysical characterization of the R12E and R12E E104R mutants together with their severe impact on IKK kinase activity and NF-κB activation.***A*, *left*, SEC MALS chromatograms obtained for WT (*black*) and R12E (*mauve*) vFLIP. The molecular weights corresponding to vFLIP monomer and dimer peaks are shown in *red* and *green*, respectively. *Right*, SEC profiles for WT vFLIP (*black*), the R12E E104R mutant (*pink*), and the R12E mutant (*green*) following the addition of 10 mM TCEP prior to loading. *B*, isothermal calorimetry (ITC) data obtained for WT vFLIP and the R12E mutant together with calculated Kd values. *C*, *top*, immunoprecipitation of the IKK kinase complex from HEK293T cells expressing WT, R12E, R12E E104R, and D102R E104R vFLIP mutants designed to disrupt the vFLIP–IKKγ and vFLIP–vFLIP interfaces, followed by immunoblotting with anti-vFLIP antibody to assess the levels of vFLIP–IKKγ complex formation. Lanes marked N correspond to immunoprecipitations performed using anti-IKKγ (Nemo) antibody and I, those performed using an isotype-matched control IgG. *Bottom*, kinase assays of WT and mutant complexes following immunoprecipitation of the IKK kinase complex from HEK293T cells expressing WT or mutant vFLIP using an anti-IKKγ antibody. These were conducted in the presence of γ^32^p-ATP, and the quantities of phosphorylated GST-IκB (1–54) (pGST-IκBα) were determined for WT vFLIP and mutants. All assays were performed in triplicate. *D*, *top*, NF-κB luciferase assays performed for HEK293 cells expressing WT and vFLIP mutants. Five independent biological replicate experiments were performed, and the data were normalized by comparison to NF-κB luciferase activity in the absence of vFLIP. A box plot of the five data points for WT and vFLIP mutants is shown. *Bottom*, one-way ANOVA testing demonstrates significant difference between these data. Post-hoc pairwise comparison demonstrates that the only significant differences are between WT vFLIP and the three vFLIP mutants. IKK, inhibitor of κB kinase.

Having identified putative vFLIP–IKKγ and vFLIP–vFLIP interfaces within higher order assemblies, both were targeted for disruption using site directed mutagenesis. An R12E vFLIP mutant was generated to maximally disrupt the vFLIP–IKKγ interactions and a D102R E104R vFLIP double mutant to disrupt vFLIP–vFLIP contacts. An R12E E104R vFLIP double mutant was also constructed to determine the effects of disrupting both regions simultaneously. Although designed to disrupt oligomerization, all mutants needed to retain their capacity to associate with IKKγ given that the heterotetramer complex is fundamental to the formation of higher order assemblies as illustrated in Figure 4*A*. Any disruption of the heterotetramer would therefore have a profound effect on oligomerization without addressing the question of its role in activation. This derives from the fact that contacts involved in oligomerization are relatively weak compared to those associated with heterotetramer formation that are largely responsible for the nanomolar Kd associated with the vFLIP–IKKγ interaction, as demonstrated by our studies involving an IKKγ mimetic stapled peptide (16). Whilst incapable of forming vFLIP–IKKγ interactions outside of the heterotetramer interface given the absence of residues implicated in oligomerization, this peptide nonetheless demonstrated a comparable affinity to IKKγ. A prerequisite for any putative oligomerization-defective mutants would therefore be an affinity for IKKγ similar to that of WT vFLIP to rule out the possibility of impaired heterotetramerization. To investigate this, the R12E, D102R E104R, and R12E E104R vFLIP mutants were expressed in *E. coli* for biochemical/biophysical analysis. All were observed to have a similar elution profile during SEC MALS to that of WT vFLIP with the exception of R12E (Fig. 5*A*). Rather than eluting predominantly as a monomer (preceded by a small dimer peak), the R12E mutant had an enhanced dimeric component comparable to that of the monomer in terms of UV absorbance (Fig. 5*A*, left). The tendency of this mutant to dimerize was confirmed by native mass spectrometry (Fig. S2*B*) and shown to be reducing agent dependent since treatment with 10 mM TCEP resulted in a shift to a predominant monomer when re-investigated using SEC (Fig. 5*A*, right). These observations could be explained by the crystal structure of the R12E mutant that was refined to 4.2 Å (Supporting Data 3 and Table S1). In this structure, six R12E monomers form the asymmetric unit, two of which appear to be appropriately positioned for formation of a disulfide bridge mediated by C106, in an extended but weakly stabilized interface that also incorporates E12 and K13 (Fig. S3). Consistent with modification of the C106 region in the R12E E104R mutant, however, an inability to form an equivalent disulfide bridge would be predicted and was confirmed by the monomeric elution profile observed for this mutant (Fig. 5*A*, right). Despite these differences, all mutants were seemingly unaffected in their capacity to form vFLIP–IKKγ (150–272) heterotetramer complexes when analyzed using SEC MALS (Fig. S4). Furthermore, ITC experiments confirmed that the R12E mutant had a similar Kd to that of WT vFLIP (Fig. 5*B*) consistent with previous reports (16) and the high levels of similarity between the R12E vFLIP and WT vFLIP co-ordinates in the vFLIP–IKKγ(150–272) complex when superposed (Fig. S5). Our data therefore illustrate that the introduction of mutations designed to disrupt oligomerization had no impact on the structural integrity of vFLIP and therefore its ability to form vFLIP–IKKγ heterotetramers.

We next investigated the capacity of the R12E, R12E E104R and D102R E104R vFLIP mutants to activate NF-κB using a combination of luciferase reporter and IκBα phosphorylation assays. Whilst all mutants appeared unimpaired in their capacity to associate with IKKγ following immunoprecipitation consistent with our biochemical and biophysical assays (Fig. 5*C*) and were expressed at similar levels, R12E displayed significant reductions in its capacity to phosphorylate GST-IκBα(1–54) and NF-κB activation (Fig. 5, *C* and *D*). More modest impairments were evident for the D102R E104R mutant. The highest levels of attenuation, however, were observed for the R12E E104R double mutant, clearly indicating that both vFLIP–IKKγ and vFLIP–vFLIP interfaces have an important role in activation.

**Figure 6:**
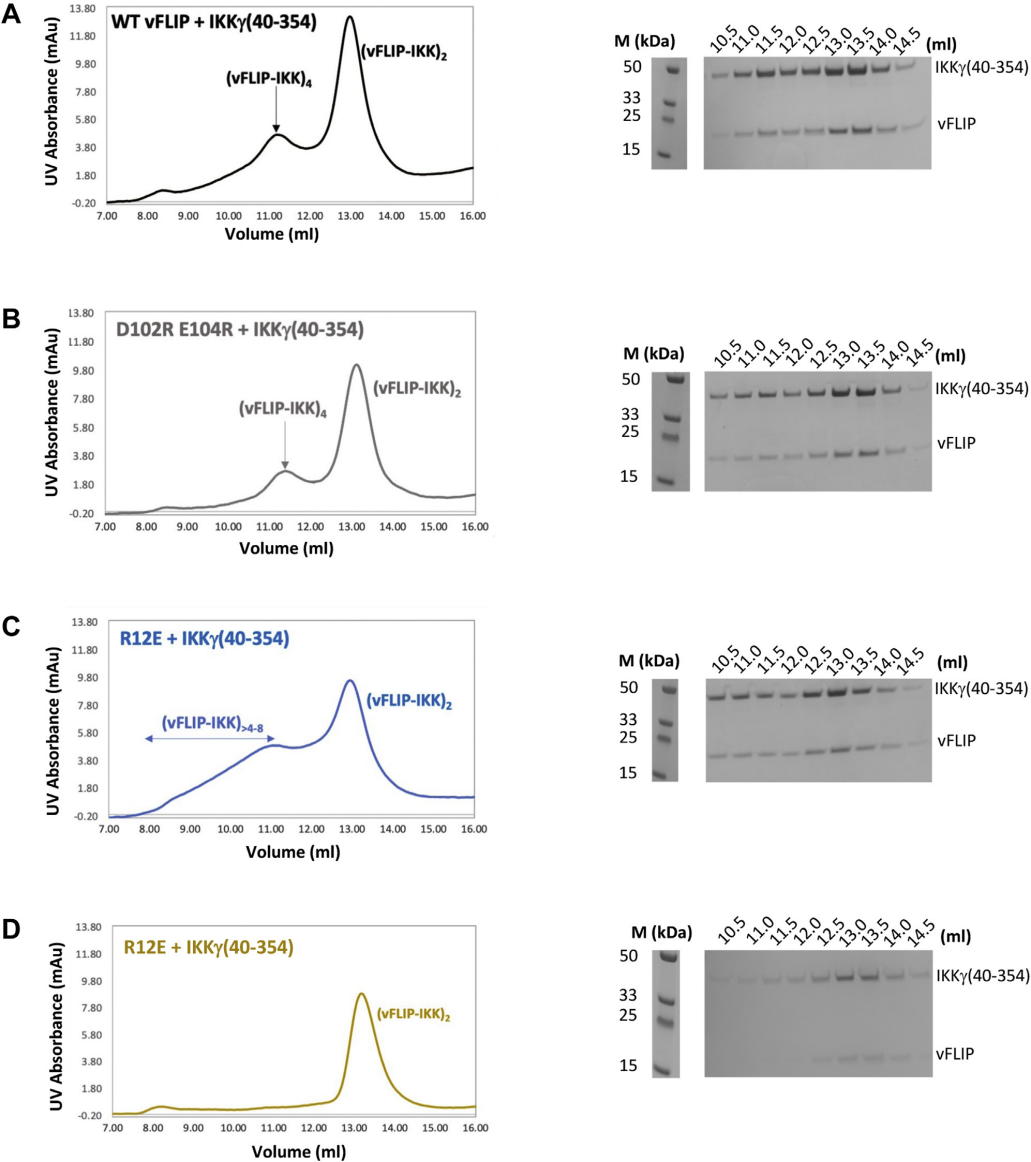
**SEC analysis of WT and mutant vFLIP–IKKγ(40–354) complexes.***Left*, chromatogram and *right*, SDS PAGE analysis of the eluted peaks. Lanes marked M correspond to those containing molecular weight markers. *A*, WT vFLIP–IKKγ(40–354). *B*, D102R E104R vFLIP–IKKγ(40–354). *C*, R12E vFLIP–IKKγ(40–354). *D*, R12E E104R vFLIP–IKKγ(40–354). IKK, inhibitor of κB kinase; SEC, size-exclusion chromatography.

**Figure 7:**
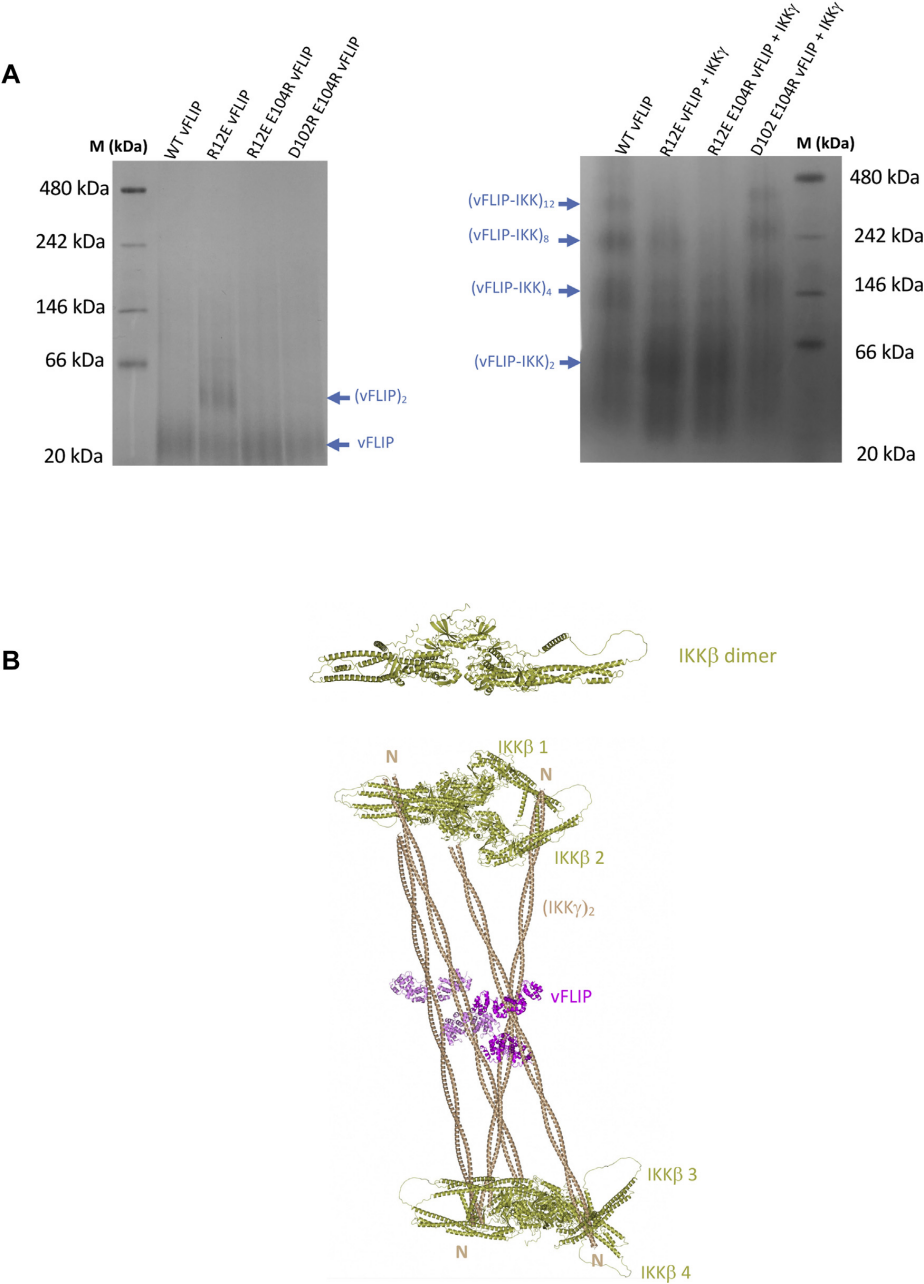
**Blue native gel analysis of mutant vFLIP-IKKγ complexes and a putative model for vFLIP-induced IKK kinase activation.***A*, *left*, blue native gel showing the migration profiles for WT, R12E, R12E E104R and D102R E104R vFLIP proteins and *right*, the associated vFLIP–IKKγ(150–272) complexes. M denotes marker lanes. *B*, schematic model of how the combination of oligomeric vFLIP–IKKγ and vFLIP–vFLIP contacts in the hetero-octamer may generate a structure into which four IKKβ ”activated” dimers can be docked. *Top*, structure of an active IKKβ dimer. This was constructed from PDB entry 4E3C as reported in (20, 21) with the final model constituting superposed AlphaFold (45)-derived monomers of full-length IKKβ. *Bottom*, putative structure of the hetero-octamer vFLIP–IKKγ complex incorporating four IKKβ dimers (labeled IKKβ1–4 in *gold*) in their active conformation. To generate this model, IKKγ(150–272) dimer co-ordinates were replaced with a model for IKKγ residues 54 to 354 derived from our previous EPR studies (15, 18) in the vFLIP–IKKγ hetero-octamer. Activated IKKβ dimers were next docked onto the N-terminal ends of neighboring IKKγ molecules (denoted by N) *via* their binding motifs as illustrated in PDB entry 3BRV (46). The IKKγ dimers are colored *wheat* and the vFLIP monomers *magenta*. EPR, electron paramagnetic resonance; IKK, inhibitor of κB kinase.

Although cell-based assays illustrated the importance of both vFLIP–IKKγ and vFLIP–vFLIP contacts, they did not address whether impaired NF-κB activation could be directly associated with defective oligomerization. To establish this, SEC was performed on all mutant complexes involving the IKKγ(40–354) construct (Fig. 6, *A*–*D*). Whilst the heterotetramer complex was predominant in all, similar to WT vFLIP, there were significant differences in the ability of the mutants to support hetero-octamer formation. Although the D102R E104R mutant was the least disrupted and had a near WT elution profile, the R12E mutant lacked a defined hetero-octamer peak that was instead replaced by a broad range of interchanging higher molecular species extending from the hetero-octamer region almost to the void of the column. By contrast, the only discernible peak for the R12E E104R mutant was that of the heterotetramer. Those corresponding to the hetero-octamer and higher order species were entirely absent. Attempts were also made to analyze the mutant IKKγ(40–354) complexes using native mass spectrometry but were largely unsuccessful. Whilst data could be obtained for the R12E mutant in complex with the IKKγ(150–272) construct, substantial reductions in the abundances of the vFLIP–IKKγ heterotetramer species were observed along with significant increases in the quantities of vFLIP alone (Fig. S6, *A* and *B*). This is likely to derive from the stability of the R12E–IKKγ complex being compromised by the necessary use of the native mass spectrometry buffer previously described. Blue native gel analysis of the mutant complexes, however, revealed similar oligomerization defects to those observed using SEC (Fig. 7*A*). The defects evident from SEC and blue native gel analysis strongly correlate with the levels of defective NF-κB activation and phosphorylation of GST- IκBα(1–54) observed in cell-based assays. Our findings thus confirm that the vFLIP–IKKγ and vFLIP–vFLIP interfaces identified in our crystal structure have a key role in vFLIP-induced NF-κB activation through the formation of hetero-octameric and higher order assemblies.

## Discussion

To date, it has been unclear how vFLIP encoded by the KSHV virus activates the canonical NF-κB pathway through its association with IKKγ, the essential modulatory element of the IKK kinase complex. In keeping with the lack of obvious co-activators, we have confirmed that vFLIP alone is sufficient for kinase complex activation through a process of autophosphorylation. We have also shown that central to this process is the formation of a vFLIP–IKKγ hetero-octamer and higher order species, stabilized by a continuous surface composed of both vFLIP–vFLIP and vFLIP–IKKγ interactions whose disruption substantially downregulates NF-κB activation.

Whilst multimeric assemblies have been postulated for the IKK kinase complex based on their potential to regulate signal transduction (31) and lattice structures generated by such species directly observed using super resolution microscopy (22, 32), ours is the first study to provide a detailed analysis of the nature of these assemblies in response to vFLIP-induced activation. The presence of these multimers at relatively low levels indicated by mass spectrometry together with CID fragmentation and PISA analysis confirms that they are weakly stabilized and are analogous to the low abundance of the reported lattice structures observed in U2OS cells (22). It has been hypothesized that this low abundance is required for efficiently balancing signal intensity with noise filtering which are pivotal to regulation of the pathway. In the case of vFLIP-induced activation that is deregulated, it can nonetheless be speculated that sustained high levels of NF-κB activation would be deleterious to the cell and ultimately to the survival and eventual proliferation of KSHV. A strategy to offset this may therefore be to maintain relatively low levels of persistent activation which is consistent with the modest increases evident in our cell-based activity assays involving vFLIP (Fig. 1).

Key to the formation of oligomeric vFLIP–IKKγ assemblies are interactions contributed by amino acids R12 and E104 in vFLIP. These residues are not conserved in the cellular FLIP homologues and would thus be unable to mediate similar contacts, in keeping with the inability of cellular FLIPs to directly associate with IKKγ and activate the IKK kinase (14). Likewise, rhesus monkey rhadinovirus vFLIP lacks key residues implicated in oligomerization and does not activate NF-κB, together with pox molluscum contagiosum virus protein MC159 that despite being able to associate with IKKγ inhibits TNF-induced NF-κB activation (33, 34).

The fact that the R12E mutant appears capable of forming higher order species based on our SEC data, the lack of a clearly defined hetero-octamer coupled with the defective oligomerization observed in blue native gels suggests that the vFLIP–IKKγ assemblies capable of activating the IKK kinase are structurally specific. This apparent requirement for higher order assemblies, though analogous to activation triggered by inflammatory stimuli, differs in being ubiquitin-chain independent. It can, however, be speculated that the role of ubiquitin chains in activation of the IKK kinase may be (at least in part) functionally equivalent to vFLIP in enabling specific oligomerization of IKKγ as a precursor to the formation of docking platforms for upstream kinases as part of a “priming” event. This priming event involves the kinase TAK1 that phosphorylates Ser 177 of IKKβ prior to autophosphorylation of Ser 181 within the same monomer (35). Such an event is not required for vFLIP-mediated IKK kinase activation that alternatively relies on autophosphorylation in *trans* based on a specific combination of vFLIP–vFLIP and vFLIP–IKKγ contacts. Manual docking experiments suggest that the vFLIP–IKKγ hetero-octamer could indeed form the repeat unit of a lattice in which activated IKKβ dimers would be both favorably accommodated and stabilized (Fig. 7*B*). This ubiquitin-independent mode of IKK kinase activation observed for vFLIP, however, contrasts with the mechanism utilized by the HTLV-1 viral oncoprotein TAX, a functional analog. This appears to be more resemblant of the cytokine-induced pathway where TAX can generate and associate with its own ubiquitin chains, by means of an intrinsic E3 ligase activity, to which IKKγ can then be recruited (36). A potential role for oligomerization and other mechanistic details, nonetheless, have yet to be ascertained for TAX-induced NF-κB activation. It is interesting to speculate, however, that IKKγ mutants shown to constitutively upregulate the canonical NF-κB pathway (37) may also bypass the need for ubiquitin chains, analogous to vFLIP, owing to an innate ability to form similar or alternative active IKK kinase multimers.

## Experimental procedures

### Cell-based assays

To determine the capacity of recombinant vFLIP to activate NF-κB and phosphorylate recombinant GST-IκBα (1–54), the IKK kinase complex was immunoprecipitated from unstimulated HEK293T cells using an anti-IKKγ antibody (FL419, Santa Cruz) following disruption in lysis buffer (20 mM Tris-HCl, pH 7.5, 150 mM NaCl, 1% Triton-X 100, 5% glycerol, 1 mM PMSF, and protease inhibitor cocktail) and then incubated with recombinant vFLIP, in the concentration range 0.15 to 4.0 μM, that had been expressed and purified as reported (15). Reactions were allowed to proceed either for 1 h at 37 °C or overnight at 4 °C, and following incubation, immune complexes were washed with kinase wash buffer (20 mM Hepes, pH 7.6, 50 mM NaCl, 20 mM β-glycerophosphate, 0.5 mM DTT, and 1 mM PMSF) and their capacity to phosphorylate recombinant GST-IκBα(1–54) using a kinase reaction buffer (20 mM Hepes, pH 7.6, 50 mM NaCl, 10 mM MgCl_2_, 2 mM DTT, 0.1 mM Na_3_VO_4_, 20 μM γ^32^ATP) determined as described in (9). In brief, following removal of the kinase wash buffer, 40 μl of kinase reaction buffer and 1 to 2 μg of recombinant GST-IκBα(1–54) together with 0.5 ml of ^32^γ-ATP were added to each sample. These were subsequently incubated at 30 °C for 30 min, and the reactions halted by the addition of 4× Lammeli buffer prior to incubation at 95 °C for 5 min. The samples were next applied to 10% SDS PAGE gels which were dried following electrophoresis prior to imaging using a phosphor screen. Kinase assays involving LPP were also performed in the presence of 3 μg of recombinant vFLIP. Immune complexes derived from cell lysates of HEK293T cells transfected with 3 μg of pCDNA3-vFLIP were washed twice with high salt wash buffer (25 mM Tris-HCl, pH 7.6, 500 mM NaCl, 1 mM EGTA, 1 mM DTT, 1% Triton-X-100, 5% glycerol, 1 mM Na_3_VO_4_, 1 mM β-glycerophosphate, 5 mM NaF, 1 mM PMSF) followed by two washes in kinase wash buffer. The immunoprecipitates were next incubated in phosphatase reaction buffer containing increasing concentrations of LPP (4, 40 and 400 U) for 30 min at 30 °C. The reactions were terminated by the addition of 25 mM Na_3_VO_4_ and 20 mM NaF. The treated complexes were subsequently washed with kinase wash buffer and their capacity to phosphorylate GST-IκBα(1–54) using γ^32^ATP determined by means of the kinase assay outlined above (a more detailed description can be found in (9)). To visualize the radiolabeled bands, phosphor screens were scanned using a Typhoon Phosphorimager (GE Healthcare), and the band intensities were quantified using ImageQuant TL 7.0 software (GE Healthcare).

NF-κB activation assays involving the T-loop IKKβ AA (IKKβ SSAA or S171/S181AA) and IKKβ EE (IKKβ SSEE or S171/S181EE) mutants were performed using the luciferase reporter system described in (9, 14). Mutants were generated as described in Mercurio *et al.* (7) with each experiment being performed at least in triplicate. NF-κB activation assays involving the oligomerization mutations were also performed using the reporter system described in (9, 14).

### Recombinant protein production and site directed mutagenesis

WT vFLIP(1–177), the IKKγ(150–272), and IKKγ(40–354) constructs were expressed and purified as previously described (15, 18).

The R12E, R12E E104R, and D102R E104R vFLIP mutants were generated using QuikChange (Agilent) and purified following the protocol for WT vFLIP.

### Blue native gel electrophoresis

Purified complexes (8–10 μg) comprising IKKγ(150–272) bound to WT vFLIP and single/double mutants or those assembled from concentrated samples of the individual proteins were loaded onto native gels (4–20%, Novagen). They were run following the manufacturer’s instructions, and experiments were performed in triplicate. To analyze the protein components of the various species, individual bands were excised following electrophoresis and soaked in sample buffer prior to analysis on a 4 to 20% BOLT SDS PAGE gel (Novagen).

### SEC MALS and SEC

For SEC MALS experiments, preformed complexes comprising IKKγ(150–272), WT vFLIP, or mutants (as well as the proteins alone) were analyzed using a Superdex 200 10/300 GL column (Cytiva) equilibrated in a buffer comprising 25 mM Tris-HCl (pH 8.5), 200 mM NaCl, and 1 mM DTT. Samples (100 μl) were applied at a concentration of 20 mg/ml for all complexes, 3 mg/ml for IKKγ (150–272), and 0.5 mg/ml for WT vFLIP and mutants since WT vFLIP precipitates at higher concentrations. Complexes involving WT vFLIP/mutants and the IKKγ(40–354) construct were assembled from concentrated samples of the individual proteins in a 1.2:1 molar ratio of vFLIP to IKKγ which were incubated in a buffer comprising 25 mM Tris-HCl (pH 8.5), 200 mM NaCl, 2 mM DTT for 30 min prior to concentration using a 0.5 ml Amicon Ultra (Merck Millipore) concentrator. Samples (100 μl) were applied to a Superose 6-Increase column (GE Healthcare) at a concentration of 3 mg/ml. Inline MALS detection was performed with a Dawn 8+ detector (Wyatt Technology Corp) coupled to a laser emitting light at a wavelength of 690 nm. Refractive index measurements were made using an Optilab T-rEX (Wyatt Technology Corp). The data were processed using Wyatt Technologies Astra 6.1 Software.

SEC experiments were performed using 980 μg of WT vFLIP (or mutants) applied to a Superdex 200 10/300 GL column (Cytiva) preequilibrated in the same buffer used for SEC MALS. Each experiment was performed independently in triplicate. Those performed with WT vFLIP or mutant complexes involving the IKKγ(40–354) mutant were carried out using a Superose 6 increase column (Cytiva) and preassembled complexes at the same ratios and concentrations used for SEC MALS detailed above.

### Mass spectrometry

Purified proteins (and complexes) were buffer exchanged into 200 mM ammonium acetate solution at pH 8.5 using 10 kDa cut-off Amicon Ultra 0.5 ml centrifugal spin filters (Merck Millipore). Samples were centrifuged six times at 12,000 rpm for 5 min at room temperature. Protein concentrations were determined using a Qubit protein assay (ThermoFisher Scientific), and samples were diluted to a concentration of 3.2 μM for native mass spectrometry analysis. Protein samples were introduced into a first-generation Synapt QToF mass spectrometer (Waters Corp) using a nanoelectrospray gold-coated borosilicate glass capillary (prepared in house). Instrument parameters were as follows: capillary voltage 1.2 kV, sampling cone voltage 55 V, source offset 2 V, backing pressure 3.2 mbar, or 5.9 mbar, trap collision energy 8 eV, transfer collision energy 6 eV, and bias 12 V. CID was performed using a trap collision energy of 28 eV and a transfer collision energy of 26 eV. Native mass spectrometry data were processed using MassLynx 4.2 (Waters Corp), UniDec and in house software Amphitrite (38, 39).

### Isothermal calorimetry

Isothermal calorimetry experiments were performed using a Microcal VP-ITC microcalorimeter with all proteins dialyzed into a buffer comprising 25 mM Tris-HCl, pH 8.5, 200 mM NaCl, and 2 mM TCEP. For the binding reactions, syringe concentrations varied between ∼85 and 100 μM for IKKγ(150–272) and cell concentrations between ∼8 and 10 μM for vFLIP. A 2 μl injection, followed by 20 10 μl injections with stirring at 300 rpm were used for the titrations. Experiments were performed at least in duplicate.

### Crystallization, data collection, structure determination, and refinement of the R12E mutant

Details can be found in Supporting Data 3 and Table S1.

## Data availability

Co-ordinates and structure factors for the R12E vFLIP mutant have been deposited at the protein data bank under the accession code 7PDJ.

## Supporting information

This article contains supporting information (40, 41, 42, 43, 44).

## Conflict of interest

The authors declare that they have no conflicts of interest with the contents of this article.

## References

[1] Chang Y., Moore P. (2014). Twenty years of KSHV. Viruses.

[2] Schulz T.F. (2000). Kaposi's sarcoma-associated herpesvirus (human herpesvirus 8): epidemiology and pathogenesis. J. Antimicrob. Chemother..

[3] Arvanitakis L., Mesri E.A., Nador R.G., Said J.W., Asch A.S., Knowles D.M. (1996). Establishment and characterization of a primary effusion (body cavity-based) lymphoma cell line (BC-3) harboring Kaposi's sarcoma-associated herpesvirus (KSHV/HHV-8) in the absence of Epstein-Barr virus. Blood.

[4] Boshoff C., Weiss R.A. (1998). Kaposi's sarcoma-associated herpesvirus. Adv. Cancer Res..

[5] Oeckinghaus A., Ghosh S. (2009). The NF-kappaB family of transcription factors and its regulation. Cold Spring Harb. Perspect. Biol..

[6] DiDonato J.A., Hayakawa M., Rothwarf D.M., Zandi E., Karin M. (1997). A cytokine-responsive IkappaB kinase that activates the transcription factor NF-kappaB. Nature.

[7] Mercurio F., Zhu H., Murray B.W., Shevchenko A., Bennett B.L., Li J. (1997). IKK-1 and IKK-2: cytokine-activated IkappaB kinases essential for NF-kappaB activation. Science.

[8] Schulz T.F., Cesarman E. (2015). Kaposi sarcoma-associated herpesvirus: mechanisms of oncogenesis. Curr. Opin. Virol..

[9] Field N., Low W., Daniels M., Howell S., Daviet L., Boshoff C. (2003). KSHV vFLIP binds to IKK-gamma to activate IKK. J. Cell Sci..

[10] Israel A. (2010). The IKK complex, a central regulator of NF-kappaB activation. Cold Spring Harb. Perspect. Biol..

[11] Delhase M., Hayakawa M., Chen Y., Karin M. (1999). Positive and negative regulation of IkappaB kinase activity through IKKbeta subunit phosphorylation. Science.

[12] Wang C., Deng L., Hong M., Akkaraju G.R., Inoue J., Chen Z.J. (2001). TAK1 is a ubiquitin-dependent kinase of MKK and IKK. Nature.

[13] Tolani B., Matta H., Gopalakrishnan R., Punj V., Chaudhary P.M. (2014). NEMO is essential for Kaposi's sarcoma-associated herpesvirus-encoded vFLIP K13-induced gene expression and protection against death receptor-induced cell death, and its N-terminal 251 residues are sufficient for this process. J. Virol..

[14] Baratchian M., Davis C.A., Shimizu A., Escors D., Bagneris C., Barrett T. (2016). et al Distinct activation mechanisms of NF-kappaB regulator IKK by isoforms of the cell death regulator cFLIP. J. Biol. Chem..

[15] Bagneris C., Ageichik A.V., Cronin N., Wallace B., Collins M., Boshoff C. (2008). Crystal structure of a vFlip-IKKgamma complex: insights into viral activation of the IKK signalosome. Mol. Cell.

[16] Briggs L.C., Chan A.W.E., Davis C.A., Whitelock N., Hotiana H.A., Baratchian M. (2017). IKKgamma mimetic peptides block the resistance to apoptosis associated with KSHV infection. J Virol..

[17] Hauenstein A.V., Xu G., Kabaleeswaran V., Wu H. (2017). Evidence for M1-linked polyubiquitin-mediated conformational change in NEMO. J. Mol. Biol..

[18] Bagneris C., Rogala K.B., Baratchian M., Zamfir V., Kunze M.B., Dagless S. (2015). Probing the solution structure of IkappaB kinase (IKK) subunit gamma and its interaction with Kaposi sarcoma-associated herpes virus Flice-interacting protein and IKK subunit beta by EPR spectroscopy. J. Biol. Chem..

[19] Matta H., Gopalakrishnan R., Graham C., Tolani B., Khanna A., Yi H. (2012). Kaposi's sarcoma associated herpesvirus encoded viral FLICE inhibitory protein K13 activates NF-kappaB pathway independent of TRAF6, TAK1 and LUBAC. PLoS One.

[20] Hauenstein A.V., Rogers W.E., Shaul J.D., Huang D.B., Ghosh G., Huxford T. (2014). Probing kinase activation and substrate specificity with an engineered monomeric IKK2. Biochemistry.

[21] Polley S., Huang D.B., Hauenstein A.V., Fusco A.J., Zhong X., Vu D. (2013). A structural basis for IkappaB kinase 2 activation via oligomerization-dependent trans auto-phosphorylation. PLoS Biol..

[22] Scholefield J., Henriques R., Savulescu A.F., Fontan E., Boucharlat A., Laplantine E. (2016). Super-resolution microscopy reveals a preformed NEMO lattice structure that is collapsed in incontinentia pigmenti. Nat. Commun..

[23] Kai X., Chellappa V., Donado C., Reyon D., Sekigami Y., Ataca D. (2014). IkappaB kinase beta (IKBKB) mutations in lymphomas that constitutively activate canonical nuclear factor kappaB (NFkappaB) signaling. J. Biol. Chem..

[24] Maubach G., Schmadicke A.C., Naumann M. (2017). NEMO links nuclear factor-kappaB to human diseases. Trends Mol. Med..

[25] Fusco F., Pescatore A., Conte M.I., Mirabelli P., Paciolla M., Esposito E. (2015). EDA-ID and IP, two faces of the same coin: how the same IKBKG/NEMO mutation affecting the NF-kappaB pathway can cause immunodeficiency and/or inflammation. Int. Rev. Immunol..

[26] Smahi A., Courtois G., Rabia S.H., Doffinger R., Bodemer C., Munnich A. (2002). The NF-kappaB signalling pathway in human diseases: from incontinentia pigmenti to ectodermal dysplasias and immune-deficiency syndromes. Hum. Mol. Genet..

[27] Ko M.S., Biswas T., Mulero M.C., Bobkov A.A., Ghosh G., Huxford T. (2020). Structurally plastic NEMO and oligomerization prone IKK2 subunits define the behavior of human IKK2:NEMO complexes in solution. Biochim. Biophys. Acta Proteins Proteom..

[28] Chowdury S.K., Katta V., Chait B.Y. (1990). Probing conformational changes in proteins by mass spectrometry. J. Am. Chem. Soc..

[29] Catici D.A., Amos H.E., Yang Y., van den Elsen J.M., Pudney C.R. (2016). The red edge excitation shift phenomenon can be used to unmask protein structural ensembles: implications for NEMO-ubiquitin interactions. FEBS J..

[30] Catici D.A., Horne J.E., Cooper G.E., Pudney C.R. (2015). Polyubiquitin drives the molecular interactions of the NF-kappaB essential modulator (NEMO) by allosteric regulation. J. Biol. Chem..

[31] Wu H. (2013). Higher-order assemblies in a new paradigm of signal transduction. Cell.

[32] Ferrao R., Li J., Bergamin E., Wu H. (2012). Structural insights into the assembly of large oligomeric signalosomes in the toll-like receptor-interleukin-1 receptor superfamily. Sci. Signal..

[33] Randall C.M., Jokela J.A., Shisler J.L. (2012). The MC159 protein from the molluscum contagiosum poxvirus inhibits NF-kappaB activation by interacting with the IkappaB kinase complex. J. Immunol..

[34] Ritthipichai K., Nan Y., Bossis I., Zhang Y. (2012). Viral FLICE inhibitory protein of rhesus monkey rhadinovirus inhibits apoptosis by enhancing autophagosome formation. PLoS One.

[35] Zhang J., Clark K., Lawrence T., Peggie M.W., Cohen P. (2014). An unexpected twist to the activation of IKKbeta: TAK1 primes IKKbeta for activation by autophosphorylation. Biochem. J..

[36] Wang C., Long W., Peng C., Hu L., Zhang Q., Wu A. (2016). HTLV-1 tax functions as a ubiquitin E3 ligase for direct IKK activation via synthesis of mixed-linkage polyubiquitin chains. PLoS Pathog..

[37] Bloor S., Ryzhakov G., Wagner S., Butler P.J., Smith D.L., Krumbach R. (2008). Signal processing by its coil zipper domain activates IKK gamma. Proc. Natl. Acad. Sci. U. S. A..

[38] Marty M.T., Baldwin A.J., Marklund E.G., Hochberg G.K., Benesch J.L., Robinson C.V. (2015). Bayesian deconvolution of mass and ion mobility spectra: from binary interactions to polydisperse ensembles. Anal. Chem..

[39] Sivalingam G.N., Yan J., Sahota H., Thalassinos K. (2013). Amphitrite: a program for processing travelling wave ion mobility mass spectrometry data. Int. J. Mass Spectrom..

[40] Winter G., Waterman D.G., Parkhurst J.M., Brewster A.S., Gildea R.J., Gerstel M. (2018). DIALS: implementation and evaluation of a new integration package. Acta Crystallogr. D Struct. Biol..

[41] Evans P.R., Murshudov G.N. (2013). How good are my data and what is the resolution?. Acta Crystallogr. D Biol. Crystallogr..

[42] Winn M.D., Ballard C.C., Cowtan K.D., Dodson E.J., Emsley P., Evans P.R. (2011). Overview of the CCP4 suite and current developments. Acta Crystallogr. D Biol. Crystallogr..

[43] McCoy A.J., Grosse-Kunstleve R.W., Adams P.D., Winn M.D., Storoni L.C., Read R.J. (2007). Phaser crystallographic software. J. Appl. Crystallogr..

[44] Smart O.S., Womack T.O., Flensburg C., Keller P., Paciorek W., Sharff A. (2012). Exploiting structure similarity in refinement: automated NCS and target-structure restraints in BUSTER. Acta Crystallogr. D Biol. Crystallogr..

[45] Tunyasuvunakool K., Adler J., Wu Z., Green T., Zielinski M., Zidek A. (2021). Highly accurate protein structure prediction for the human proteome. Nature.

[46] Rushe M., Silvian L., Bixler S., Chen L.L., Cheung A., Bowes S. (2008). Structure of a NEMO/IKK-associating domain reveals architecture of the interaction site. Structure.

