# Supplementary material for: Mechanistic insights into the activation of the IKK kinase complex by the Kaposi’s sarcoma herpes virus oncoprotein vFLIP
Source: J Biol Chem. 2022 May 5;298(6):102012. doi: 10.1016/j.jbc.2022.102012 (PMC9163697; doi:10.1016/j.jbc.2022.102012)
Supplement: Supplemental Figures S1–S6, Supporting Data 3 and Table S1 [file mmc1.docx]

**Supporting Information**

**Mechanistic insights into the activation of the IKK kinase complex by the Kaposi’s Sarcoma Herpes virus oncoprotein vFLIP.**

Authors: Claire Bagnéris^1*^, Swathi L Senthil Kumar^1*^, Mehdi Baratchian^2^, Hannah M Britt^3^, Tufa E Assafa^4^, Konstantinos Thalassinos^3^, Mary K Collins^5^ and Tracey E Barrett^1,**^.

^1^Department of Biological sciences, Institute of Structural Molecular Biology, Birkbeck College, Malet Street, London WC1E 7HX, UK. ^2^Genitourinary Malignancies Research Center, Lerner Research Institute, Cleveland Clinic, 9500 Euclid Avenue, NB21, Cleveland, OH 44195, USA. ^3^Institute of Structural and Molecular Biology, Division of Biosciences, University College London, London, WC1E 6BT, UK. ^4^Chemistry and Biochemistry Department, University of California Santa Cruz, 1156 High Street, Santa Cruz, CA95064, USA.^5^Okinawa Institute of Science and Technology, Graduate University, 1919-1, Tancha, Onna-son, Okinawa, 904-0495 Japan.

^*^Authors who contributed equally

^**^ Corresponding author: t.barrett@bbk.ac.uk

**Figure S-1:** Native mass spectra obtained for the WT-vFLIP-IKKγ(150-272) complex together with CID fragmentation data for the hetero-tetramer and hetero-octamer species.

**Figure S-2:** Native mass spectra obtained for IKKγ(150-272) and the R12E vFLIP mutant.

**Supporting Data 3:** Crystallisation, structure determination and refinement procedures for the R12E mutant.

**Table S1:** Data collection and Refinement parameters for the final R12E model.

**Figure S-3:** Structure of the R12E vFLIP hexamer and interface linking E12 and R104 in a dimer.

**Figure S-4:** SEC MALS data obtained for IKKγ(150-272) and mutant complexes.

**Figure S-5:** Superposition of the WT-vFLIP-IKKγ co-ordinates with those of the R12E mutant in the vicinity of the IKKγ binding region.

**Figure S-6:** Native mass spectrum obtained for the R12E-vFLIP-IKKγ(150-272) complex and an analysis of the relative abundances of the various species observed compared to the WT vFLIP complex.

**
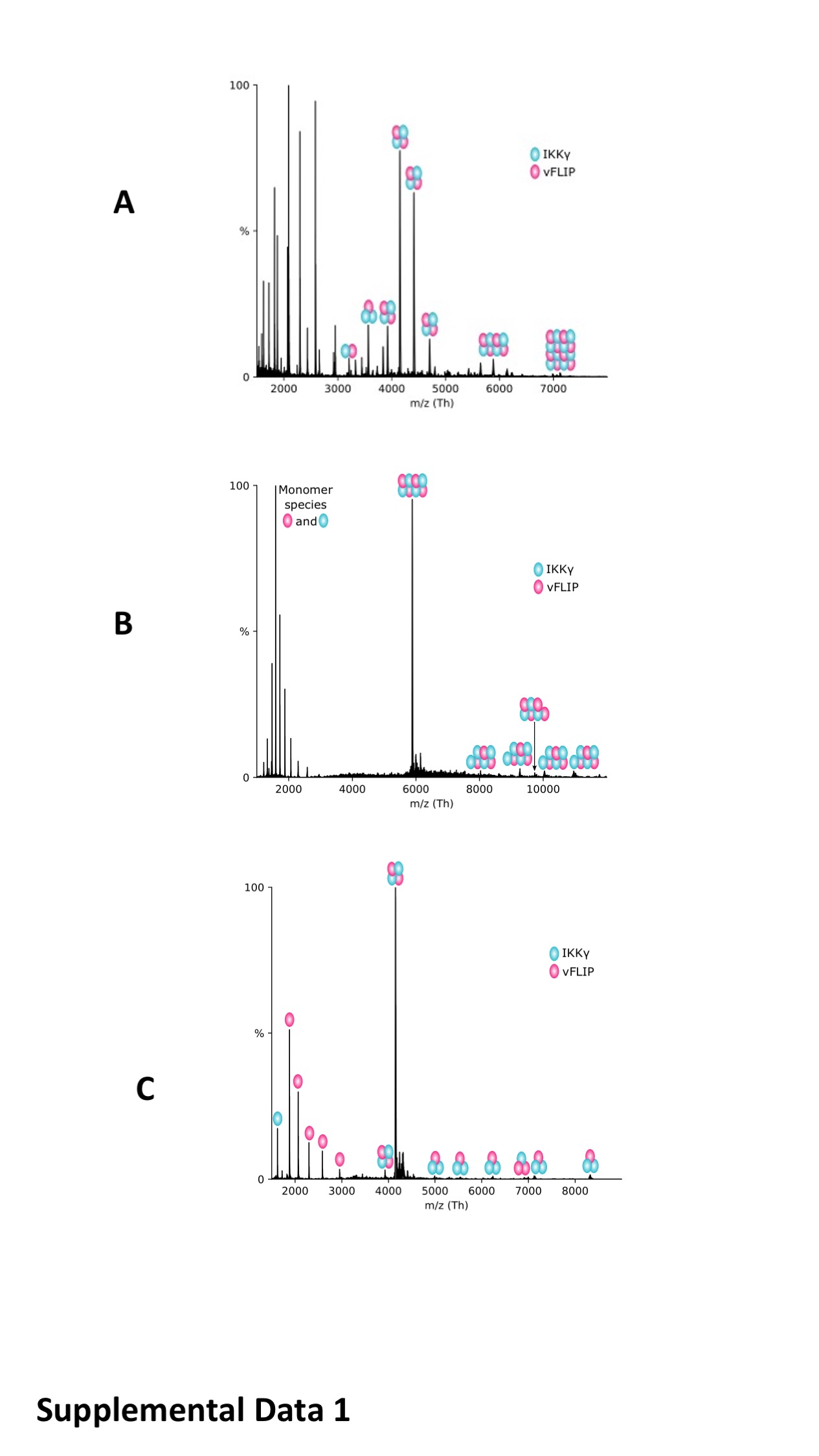
**

**Figure S-1:** Native Mass spectra for **(A)** WT-vFLIP-IKKγ showing all species and following collision induced dissociation (CID) fragmentation of **(B)** the hetero-octamer **(C)** the hetero-tetramer.

**
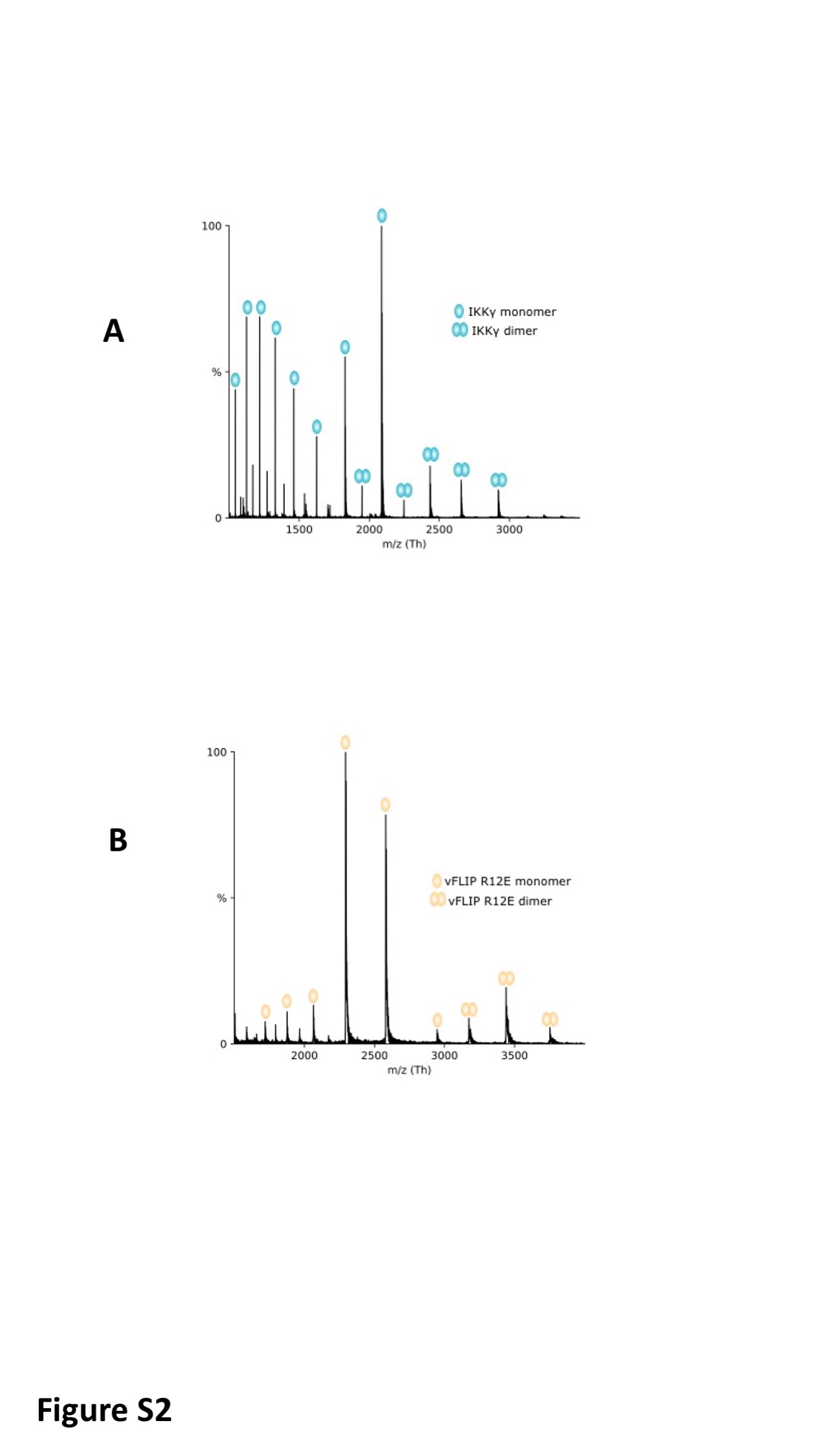
**

**Figure S-2:** Native mass spectra for **(A)** IKKγ(150-272) and **(B)** R12E vFLIP.

**Supporting Data 3**

**Crystallisation of the R12E vFLIP mutant and structure determination**

A Mosquito^TM^ crystallization robot was used to set up vapour diffusion crystallisation trials involving a range of commercially available screens and R12E vFLIP at a concentration of 9.8 mg/ml. 200 nL drops (final volume) were dispensed in a 1:1 ratio of complex to well solution and after 1 week, crystals were observed in the condition 0.1 M Sodium HEPES pH 7.0, 8 % w/v PEG 8000 (Proplex, Molecular Dimensions Ltd.). Optimal crystals were obtained from the condition 0.125 M Na HEPES pH 7.0, 1 % PEG 8000, 3% methanol and were subsequently cryo-protected in 20% ethylene glycol prior to flash freezing and irradiation at the Diamond light source on beamline i24. Images were collected to a maximum resolution of 4.2 Å on a single crystal which were integrated and scaled using DIALS (1). The data were subsequently merged and truncated using AIMLESS (CCP4 (2,3)) in space group C2. The structure was determined using molecular replacement in PHASER (4) and the vFLIP co-ordinates from protein data bank entry 3CL3, where 6 monomers were identified in the asymmetric unit. Refinement was performed using AUTOBUSTER (5) which incorporated NCS restraints and TLS refinement interspersed with cycles of manual re-building in COOT (CCP4 (2,3)). Data collection statistics and those for the refined model can be found in Table 1. The co-ordinates have been deposited at the protein data bank under the accession code 7PDJ.

**Table S1. Data collection, processing and refinement statistics.**

| **Space group** | C2 |
| --- | --- |
| **Unit cell [a, b, c (Å)]** | a= 119.40, b=69.32, c=131.89 α= 90, β=89.85 γ=90 |
| **Resolution (Å)** | 131.9-4.2 (4.7-4.2) |
| **Total no. of reflections** | 50114 |
| **No. of unique reflections** | 8076 |
| **Redundancy** | 6.2 (6.1) |
| **Completeness (%)** | 99.7 (99.2) |
| **<I>/<σ(I)>** | 2.5 (1.4) |
| ***R*_meas_^a^** | 0.21 (5.0) |
| **CC_1/2_**  **Refinement** | 0.5 (0.2) |
| **Resolution (Å)** | 66-4.2 (4.2-4.5) |
| **No. of protein/peptide atoms** | 3437 |
| **Completeness (%)** | 99.7 (99.2) |
| ***R*work^b^/*R*free^c^ (%)** | 22.7/27.3 |
| **Estimated co-ordinate error (Å)** | 0.66 |
| **Deviations from ideal stereochemistry** |  |
| **RMSD bonds (Å)** | 0.009 |
| **RMSD angles (°)** | 1.05 |
| **Wilson B-factor (Å^2^)** | 75.1 |
| **Ramachandran plot analysis^d^** |  |
| **Most Favoured (%)** | 91 |
| **Disallowed (%)** | 1.81 |
| **Table S1 continued** |  |

Values in parentheses are for the highest resolution shell (4.2– 4.7 Å).

^a^R_meas_=Σ((N/N-1))^1/2^(|Ii – <I>|)/Σ(<I>)], where the sum is calculated over all observations of a measured reflection (Ii), <I> is the mean intensity of all the measured observations (Ii), and N the total number of observations for each reflection.

^b^R_work_=Σ (|F_obs_ – F_calc_|)/Σ (F_obs_), F_obs_ are the observed structure factor amplitudes, and F_calc_ those calculated from the model.

^c^R_free_ is equivalent to R_work_ but where 5% of the measured reflections have been excluded from refinement and set aside for cross-validation purposes.

^d^Ramachandran plot analysis was from molprobity (<http://molprobity.biochem.duke.edu/>).


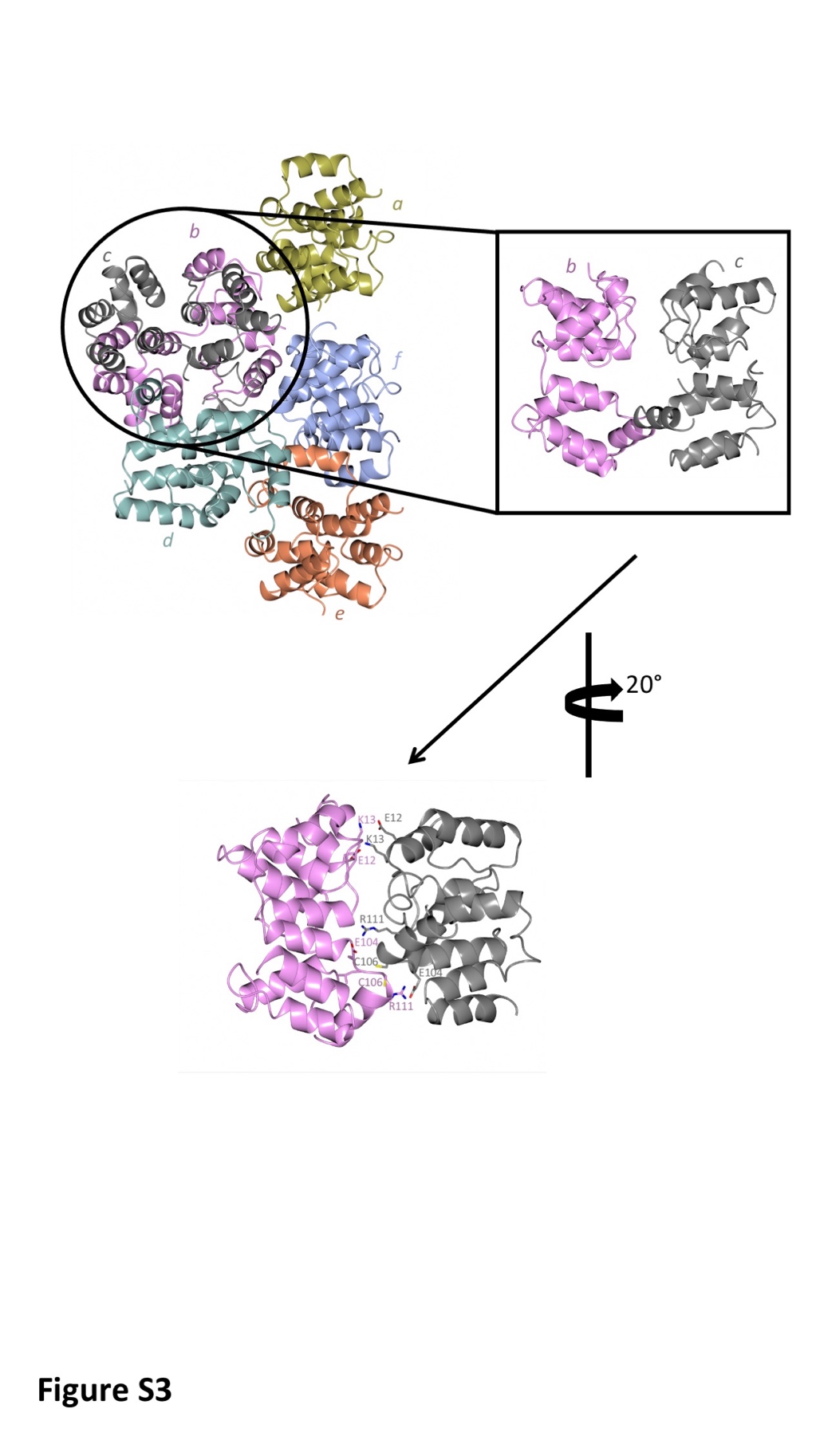


**Figure S3:** Cartoon depiction of the R12E vFLIP hexamer (monomers labelled a to f) forming the asymmetric unit with the dimer highlighted that links the E12 and E104 sites. This dimer is rotated by ~20 degrees in the lower panel to illustrate the residues involved in stabilisation.


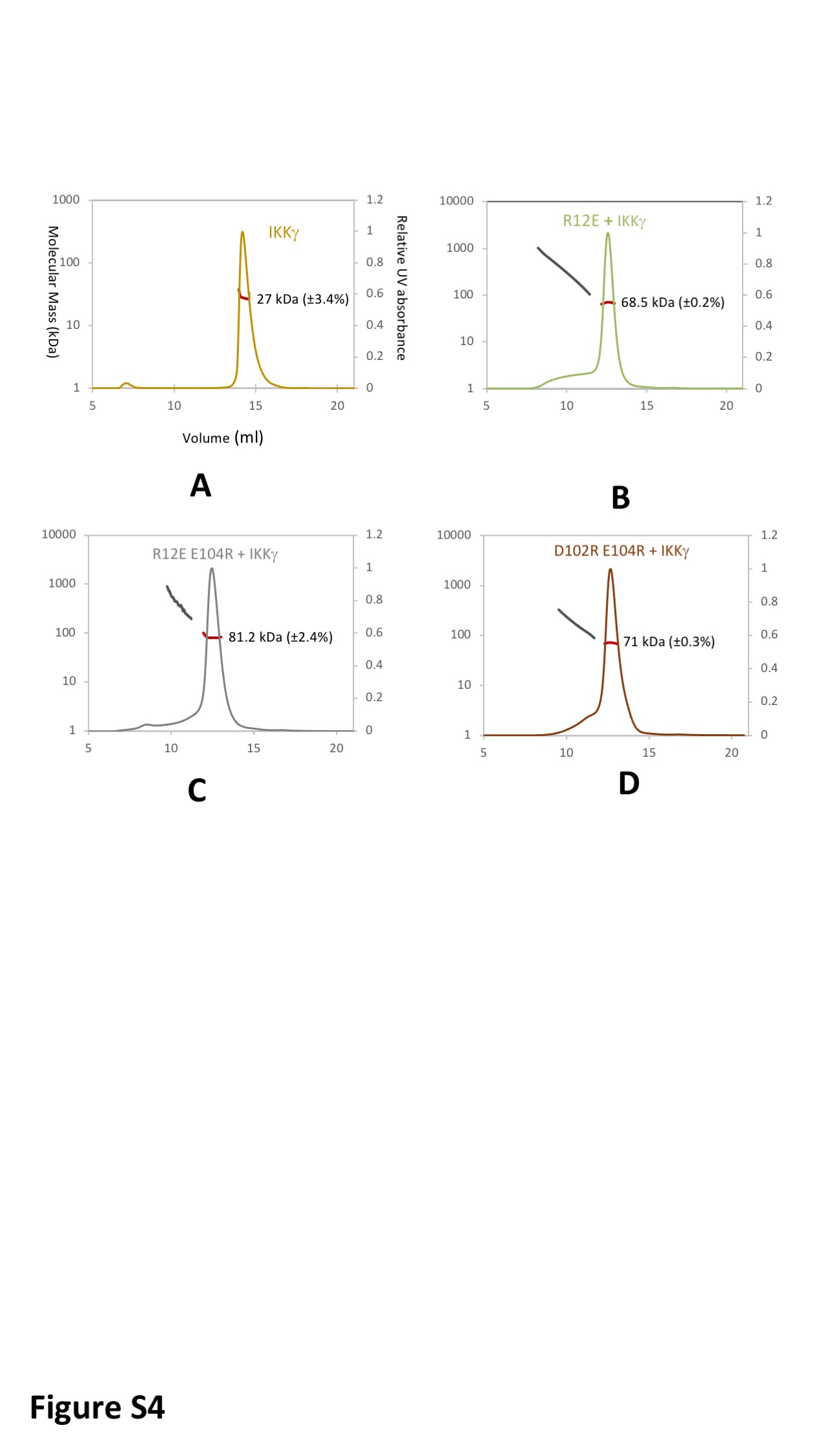


**Molecular Mass (Da)**

**A**

**Volume (ml)**

**Relative UV Absorbance**


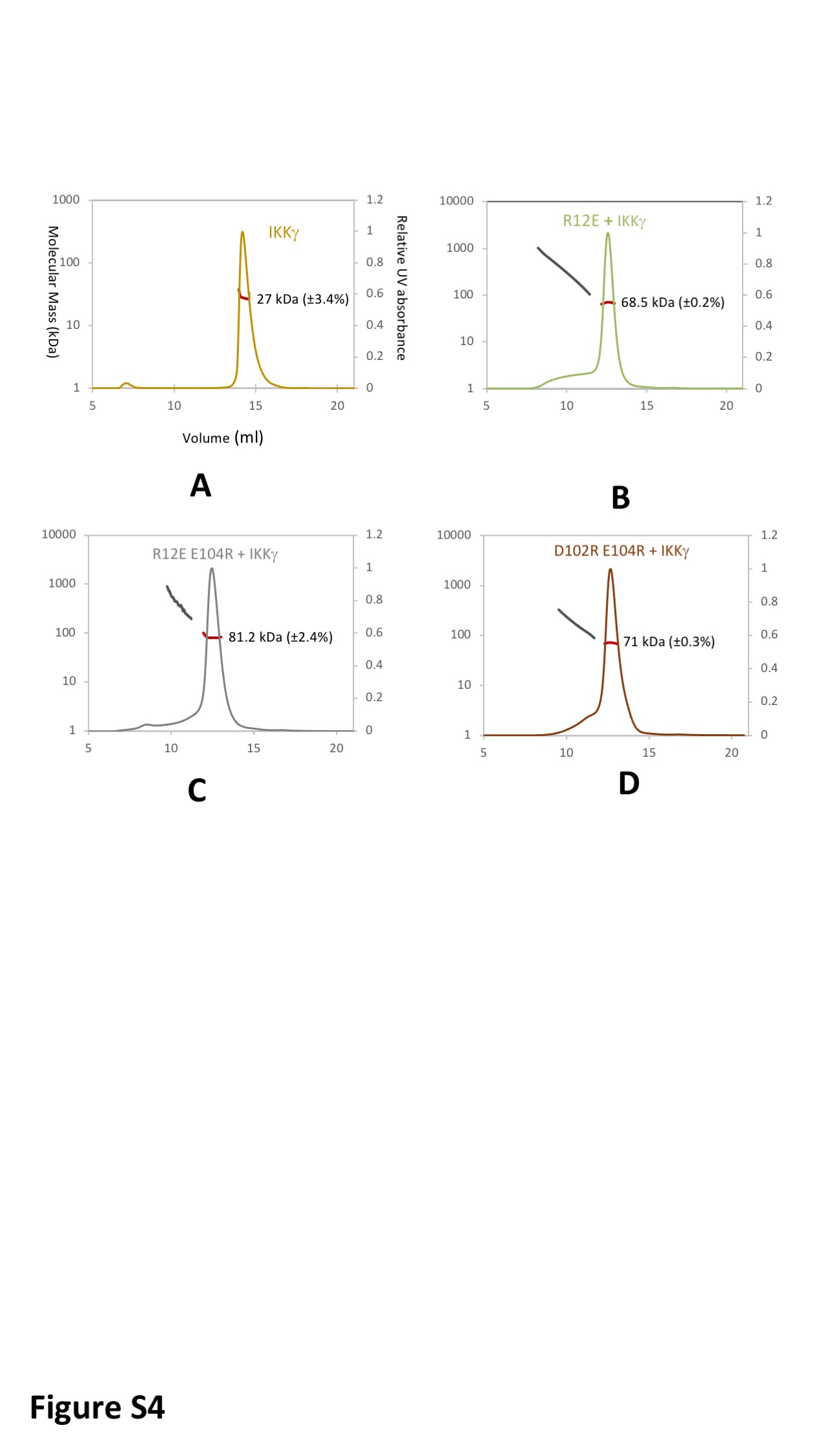


**C**

**B**

**Figure S-4:** SEC MALS chromatograms for the vFLIP-IKKγ(150-272) complexes. **(A)** R12E-IKKγ **(B)** R12E E104R-IKKγ and **(C)** D102R E104R-IKKγ. In **(A)** and **(C)**, grey lines correspond to heterogeneous high molecular species whilst those in red, indicate the vFLIP-IKKγ hetero-tetramer.

**B**


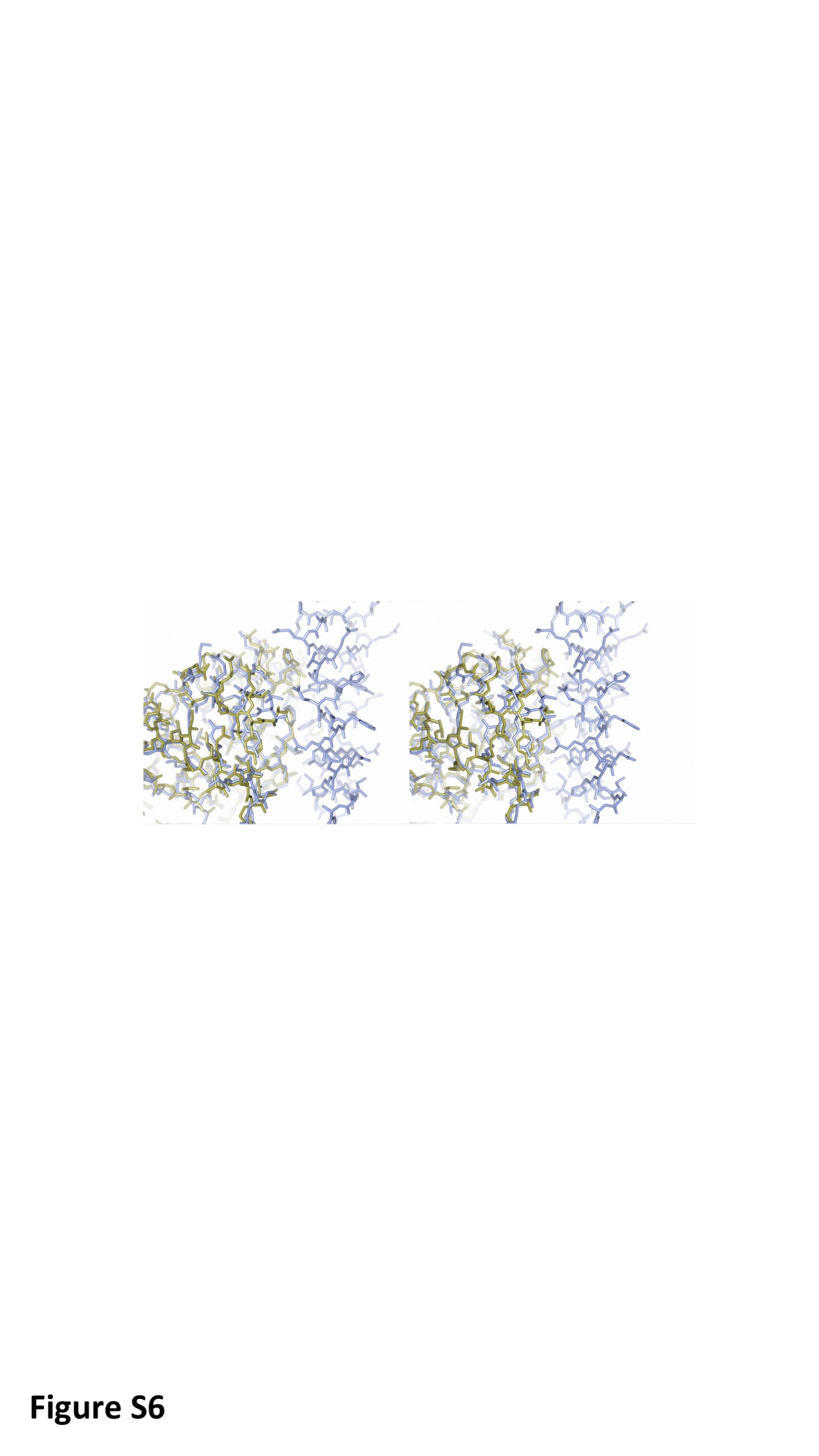


**Figure S-5:** Stereoview of the superposition of the R12E vFLIP co-ordinates (PDB accession code 7PDJ, gold) on those of the WT-vFLIP-IKKγ complex (PDB accession code 3CL3, ice).


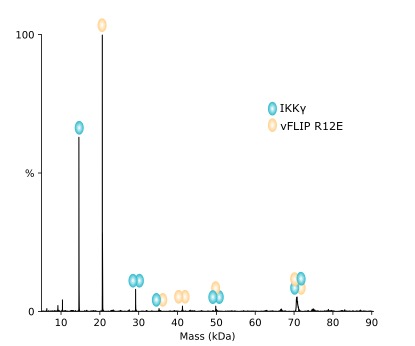


**A**

**B**


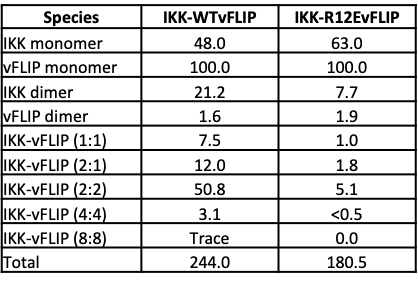


**Figure S-6: (A).** De-convoluted native mass spectrum for the R12EvFLIP-IKKγ(150-272) complex. (**B)** Table showing the abundances of the various oligomeric species for the WT and R12EvFLIP-IKKγ(150-272) complexes scaled relative to the largest component (vFLIP) which was taken as 100%.

**References**

1. Winter, G., Waterman, D. G., Parkhurst, J. M., Brewster, A. S., Gildea, R. J., Gerstel, M., Fuentes-Montero, L., Vollmar, M., Michels-Clark, T., Young, I. D., Sauter, N. K., and Evans, G. (2018) DIALS: implementation and evaluation of a new integration package. *Acta Crystallogr D Struct Biol* **74**, 85-97

2. Evans, P. R., and Murshudov, G. N. How good are my data and what is the resolution? *Acta Crystallogr D Biol Crystallogr* **69**, 1204-1214

3. Winn, M. D., Ballard, C. C., Cowtan, K. D., Dodson, E. J., Emsley, P., Evans, P. R., Keegan, R. M., Krissinel, E. B., Leslie, A. G., McCoy, A., McNicholas, S. J., Murshudov, G. N., Pannu, N. S., Potterton, E. A., Powell, H. R., Read, R. J., Vagin, A., and Wilson, K. S. Overview of the CCP4 suite and current developments. *Acta Crystallogr D Biol Crystallogr* **67**, 235-242

4. McCoy, A. J., Grosse-Kunstleve, R. W., Adams, P. D., Winn, M. D., Storoni, L. C., and Read, R. J. (2007) Phaser crystallographic software. *J Appl Crystallogr* **40**, 658-674

5. Smart, O. S., Womack, T. O., Flensburg, C., Keller, P., Paciorek, W., Sharff, A., Vonrhein, C., and Bricogne, G. Exploiting structure similarity in refinement: automated NCS and target-structure restraints in BUSTER. *Acta Crystallogr D Biol Crystallogr* **68**, 368-380
